# Dual targeting of FGFR3 and ERBB3 enhances the efficacy of FGFR inhibitors in FGFR3 fusion-driven bladder cancer

**DOI:** 10.1186/s12885-022-09478-4

**Published:** 2022-05-02

**Authors:** Andrew J. Weickhardt, David K. Lau, Margeaux Hodgson-Garms, Austen Lavis, Laura J. Jenkins, Natalia Vukelic, Paul Ioannidis, Ian Y. Luk, John M. Mariadason

**Affiliations:** 1 Olivia Newton-John Cancer and Research Institute, Melbourne, VIC Australia; 2 School of Cancer Medicine, La Trobe University, Melbourne, VIC Australia; 3 Department of Medical Oncology, Austin Health, Olivia Newton-John Cancer Wellness and Research Centre, Melbourne, VIC Australia; 4 Department of Medicine, Austin Health, University of Melbourne, Melbourne, VIC Australia

**Keywords:** Bladder cancer, FGFR3, EGFR, ERBB2, ERBB3, Targeted therapy, Acquired resistance

## Abstract

**Background:**

Mutations and fusions in Fibroblast Growth Factor Receptor 3 (*FGFR3)* occur in 10–20% of metastatic urothelial carcinomas and confer sensitivity to FGFR inhibitors. However, responses to these agents are often short-lived due to the development of acquired resistance. The objective of this study was to identify mechanisms of resistance to FGFR inhibitors in two previously uncharacterised bladder cancer cell lines harbouring *FGFR3* fusions and assess rational combination therapies to enhance sensitivity to these agents.

**Methods:**

Acquired resistance to FGFR inhibitors was generated in two *FGFR3* fusion harbouring cell lines, SW780 (*FGFR3-BAIAP2L1* fusion) and RT4 (*FGFR3-TACC3* fusion), by long-term exposure to the FGFR inhibitor BGJ398. Changes in levels of receptor tyrosine kinases were assessed by phospho-RTK arrays and immunoblotting. Changes in cell viability and proliferation were assessed by the Cell-Titre Glo assay and by propidium iodide staining and FACS analysis.

**Results:**

Long term treatment of *FGFR3*-fusion harbouring SW780 and RT4 bladder cancer cell lines with the FGFR inhibitor BGJ398 resulted in the establishment of resistant clones. These clones were cross-resistant to the clinically approved FGFR inhibitor erdafitinib and the covalently binding irreversible FGFR inhibitor TAS-120, but remained sensitive to the MEK inhibitor trametinib, indicating resistance is mediated by alternate activation of MAPK signalling. The FGFR inhibitor-resistant SW780 and RT4 lines displayed increased expression of pERBB3, and strikingly, combination treatment with an FGFR inhibitor and the ATP-competitive pan-ERBB inhibitor AZD8931 overcame this resistance. Notably, rapid induction of pERBB3 and reactivation of pERK also occurred in parental *FGFR3* fusion-driven lines within 24 h of FGFR inhibitor treatment, and combination treatment with an FGFR inhibitor and AZD8931 delayed the reactivation of pERBB3 and pERK and synergistically inhibited cell proliferation.

**Conclusions:**

We demonstrate that increased expression of pERBB3 is a key mechanism of adaptive resistance to FGFR inhibitors in FGFR3-fusion driven bladder cancers, and that this also occurs rapidly following FGFR inhibitor treatment. Our findings demonstrate that resistance can be overcome by combination treatment with a pan-ERBB inhibitor and suggest that upfront combination treatment with FGFR and pan-ERBB inhibitors warrants further investigation for *FGFR3*-fusion harbouring bladder cancers.

**Supplementary Information:**

The online version contains supplementary material available at 10.1186/s12885-022-09478-4.

## Introduction

Urothelial bladder cancer is responsible for approximately 150,000 deaths per year worldwide, and the median survival of patients with metastatic disease is approximately 18 months [1, 2]. Fibroblast Growth Factor Receptor 3 (FGFR3) is an attractive therapeutic target in bladder cancer given the 10–30% prevalence of *FGFR3* aberrations (activating mutations or aberrant gene fusions) in these tumours, and their preclinical sensitivity to FGFR-targeted therapy [3].

*FGFR3* aberrations lead to oncogenic signalling through the MAPK and PI3K pathways. Activating mutations in *FGFR3* occur in 10–20% of muscle-invasive bladder cancers [4–9] and a higher proportion in superficial and upper urinary tract urothelial cancers [10]. These mutations cluster in hotspots within exons 7, 10, and 15 of the *FGFR3* gene, with 5 mutations, R248C, S249C, G372C, Y375C, and K652E accounting for greater than 90% of all mutations [5, 8, 11, 12]. These mutations induce ligand-independent receptor dimerisation, transactivation, and constitutive activation of downstream signalling [13–15].

A smaller proportion of bladder cancers (3–6%) have *FGFR3* chromosomal translocations which generate oncogenic *FGFR3* fusion proteins [4, 16, 17]. These fusion proteins comprise of amino acids 1–760 of *FGFR3* (which include the kinase domain) fused in-frame to either transforming acid coiled-coil 3 (*TACC3*) or BAI-Associated Protein 2-Like-1 (*BAIAP2L1*) [5], and form overexpressed, permanently dimerised inclusion bodies in the cytosol that do not undergo lysosomal degradation, and are not susceptible to feedback inhibition [17]. The *FGFR3* component of the fusion gene is identical, with a conserved breakpoint lacking only the final exon (exon 19). Expression of these fusion proteins in normal human urothelial cells has been shown to induce mitogenic activation of the MAPK pathway [18].

Preclinical studies have demonstrated that human bladder cancer cell lines with *FGFR3* mutations and fusions are sensitive to FGFR inhibitors such as BGJ398 (Infigratinib, Novartis), PD173074 (Pfizer) and erdafitinib (Balversa, Janssen) [17, 19–22], thereby forming the basis for clinical trials of FGFR inhibitors in patients with metastatic urothelial cancer. Results from phase I and II trials of BGJ398 and erdafitinib in this population reported response rates of 25–40%, and based on these findings, erdafitinib was FDA approved for *FGFR2/3* aberrant bladder cancers in 2019 [23]. Despite this success, the efficacy of single agent FGFR inhibitors is limited by the short duration of efficacy with a median progression-free survival of 3.7–5.5 months [23, 24]. Several studies have investigated the mechanisms driving inherent and acquired resistance to FGFR inhibitors. However, studies in models of *FGFR3*-fusion harbouring bladder cancer lines have so far been limited to a single cell line, RT-112, which harbours a *FGFR3-TACC3* fusion as well as an amplification [25–27]. Mechanisms of resistance described in this model include epithelial-to-mesenchymal transition [25], activation of EGFR [26], ERBB2, and ERBB3 [25], and increased activation of AKT [27]. However, whether these mechanisms extend to other *FGFR3*-fusion driven bladder cancer cell lines is unknown.

To address this, we undertook this preclinical study to investigate potential resistance mechanisms to FGFR inhibitors in two previously uncharacterised bladder cancer cell lines harbouring *FGFR3*-fusions, RT4 (*FGFR3-TACC3* fusion) and SW780 (*FGFR3-BAIP2L1* fusion). Through continuous culture in the presence of the FGFR inhibitor BGJ398, we derived lines highly resistant to BGJ398. These cell lines were also found to be cross-resistant to erdafitinib (Balversa) and TAS-120 futibatinib, an irreversible FGFR inhibitor that is currently undergoing phase II clinical testing [28]. Profiling of receptor tyrosine kinases revealed increased pERBB3 in both cell lines with acquired resistance to FGFR inhibition, and we demonstrated that combination treatment with the pan-ERBB inhibitor AZD8931 (Sapitnib) can re-sensitise these cell lines to FGFR inhibitors. We also demonstrate that pERK and pERBB3 are rapidly reactivated in FGFR3-driven cell lines following FGFR inhibitor treatment, which could also be overcome by pan-ERBB receptor blockade. These findings suggest combination treatment with an FGFR and pan-ERBB inhibitor from the outset may represent a more effective approach for treating *FGFR3*-fusion driven bladder cancers.

## Methods

### Cell lines, culture and reagents

The urothelial carcinoma cell lines SW780 (*FGFR3-BAIP2L1* fusion) and RT4 (*FGFR3-TACC3* fusion) were obtained from the American Type Culture Collection (ATCC). Cells were maintained in Dulbecco’s Modified Eagle Medium (DMEM/F-12, plus glutamine and sodium bicarbonate (Invitrogen, Carlsbad, CA, USA) supplemented with 1% GlutaMax, 1% HEPES, 1% Penicillin/Streptomycin, and 10% Fetal Bovine Serum (Invitrogen), and incubated at 37 °C in 5% Carbon Dioxide. BGJ398 was obtained from Novartis (Basel, Switzerland) or from Selleck Chemicals (Houston, TX, USA). AZD8931, erdafitinib and TAS-120 were purchased from Selleck Chemicals.

### Establishment of drug resistant cell lines

Resistance to BGJ398 in SW780 and RT4 bladder cancer cell lines was established by (1) sustained exposure to 1 µM BGJ398 for 3 months (labelled SW780 RS and RT4 RS), with fresh drug added each time the cells were passaged, and (2) gradual increase in dose exposure to BGJ398 over 3 months, commencing at a low dose (3 nM) and doubling the dose each week until reaching 1 µM at which dose cells were subsequently maintained (labelled SW780 RD and RT4 RD). Parental cell lines were passaged in parallel in equivalent concentrations of DMSO. Parental and resistant cells were regularly assessed for Mycoplasma contamination and confirmed to be negative, and the authenticity of the cell lines verified using the Promega StemElite ID System (Supplementary Table 1).

### Cell viability assays

Cell viability was measured using the CellTitre-Glo (CTG) luminescent cell viability assay (Promega, Madison, WI, USA). Cells were seeded in white 96 well flat bottom plates at a density of 1500–5000 cells per well, then treated the following day with drug for 72 h. Luminescence was measured using a SpectraMax L Microplate Reader (Molecular Devices, Sunnyvale, CA, USA) and compared to DMSO treated cells. BGJ398, erdafitinib, TAS-120, Trametinib and AZD8931 were all purchased from Selleck Chemicals and dissolved in DMSO.

### Cell cycle and apoptosis assays

Changes in cell cycle kinetics and apoptosis were assessed following 24 h of drug treatment using Propidium Iodide staining as described previously [29], followed by FACS analysis using a BD FACS Canto II flow cytometer (BD Biosciences San Jose, CA). The percentage of apoptotic cells was determined by calculating the proportion of cells with a sub-diploid DNA content using the FLOWJO software V10.0 (FlowJO LLC, Ashland, OR, USA).

### Phospho-receptor tyrosine kinase (RTK) arrays

RT4-RS and SW780-RS cells were cultured in fresh media without drug for 24 h before collection to negate effects induced by acute drug exposure. Control and resistant lines were then lysed in Radio immunoprecipitation assay (RIPA) buffer (Sigma-Aldrich, St Louis, MO, USA) containing complete Protease Inhibitor Cocktail Tablets (Roche, Basel, Switzerland) and PhosSTOP (Roche). A total of 200 µg of lysate was then incubated with human Phospho-RTK Arrays (ARY001B, R&D Systems, Minneapolis, MN, USA) as per manufacturer’s instructions, and blots were read using the ChemiDoc X Imaging System (Bio-Rad, Hercules, CA, USA).

### Immunoblotting

Protein lysates were prepared as above, denatured using 10 × NuPage Sample Reducing Agent (ThermoFisher, Waltham, MA, USA), and run on NuPAGE Novex 4–12% Bis–Tris precast gels (Invitrogen) in MES (2-(*N*-morpholino) ethanesulfonic acid) buffer (ThermoFisher). Proteins were transferred using the iBlot® Dry Blotting System (Invitrogen) and signal detected using the Li-Cor® Odyssey Infrared Imager (Li-Cor, Lincoln, NE, USA). The following antibodies were obtained from Cell Signaling Technologies (Danvers, MA, USA): pERK p44/42 MAPK T202/Y204 (9106); t-ERK p44/42 MAPK (9107), p-ERBB3 Tyr1289 (2842S), ERBB3 (4754S), p-ERBB2 Tyr 1248 (2247), ERBB2 (2242), pEGFR Tyr1068 (D7A5) and EGFR (2232). Anti-β-tubulin (ab6046) was obtained from Abcam (Cambridge, UK) and anti-β-actin (A5316) from Sigma.

### Statistical analysis

Statistical analyses were performed using Prism v5 (GraphPad Software, La Jolla, CA, USA). Data shown is mean ± SEM from 3 technical replicates from a representative experiment unless stated otherwise. Biological replicates were performed for the majority of experiments and are stated in the figure legends. Groups were compared using parametric unpaired Student’s t-test with Welch’s correction. *P*-values ≤ 0.05 were considered to be statistically significant. The effect of drug combinations on cell growth in vitro was assessed using the BLISS synergy and antagonism model in the Combenefit software.

## Results

### Generation of bladder cancer cell lines with acquired resistance to FGFR inhibitors

**Fig. 1:**
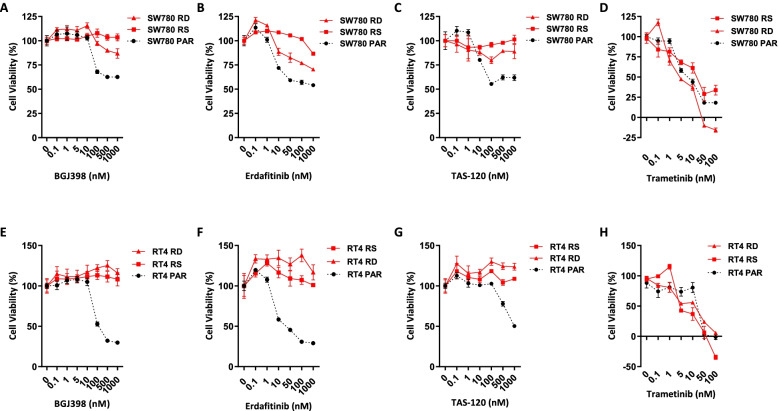
Sensitivity of SW780 (**A**-**D**) and RT4 (**E**–**H**) parental (PAR) and FGFR inhibitor resistant cell lines (SW780-RS, SW780-RD, RT4-RS, RT4-RD) to BGJ398 (**A**, **E**), erdafitinib **(B**, **F**), TAS-120 (**C**, **G**) and trametinib (**D**, **H**). Cells were treated with BGJ398, erdafitinib, TAS-120 or trametinib for 96 h and cell viability determined using the Cell Titre-Glo assay. Values shown are mean ± SEM of a representative experiment performed in triplicate

SW780 and RT4 cell lines with acquired resistance to the FGFR inhibitor BGJ398 were generated by continuous culture in 1 µM BGJ398 for 3 months, a dose which is approximately twofold higher than the clinically reported C_max_ of BGJ398 [30]. The cell lines were named SW780-RS and RT4-RS and were subsequently maintained at 1 µM BGJ398. Response of the resistant cell lines to BGJ398 was subsequently assessed by CTG assay, which confirmed that SW780-RS and RT4-RS cells were significantly more resistant to BGJ398 than matched parental control cell lines that had been cultured in parallel. Similar resistance was demonstrated in SW780-RD and RT4-RD cells lines, generated by a graduated increase in exposure to BGJ398 (Fig. 1A, E). To determine whether resistance to BGJ398 resulted in cross-resistance to other FGFR inhibitors, including erdafitinib which was recently approved for *FGFR2/3* driven bladder cancers, and the covalently binding irreversible FGFR inhibitor TAS-120 (futibatinib) [31] which is currently in early-phase clinical trials, sensitive and resistant clones were treated with increasing doses of both agents and growth inhibition assessed by CTG assay. As shown in Fig. 1, SW780 and RT4 clones that were resistant to BGJ398 were also found to be cross-resistant to erdafitinib (Fig. 1B, F) and TAS-120 (Fig. 1 C, G). Comparatively, all clones responded similarly to the MEK inhibitor trametinib (Fig. 1D, H), indicating resistance was specific to FGFR inhibitors.

### Investigation of the mechanisms of acquired drug resistance

**Fig. 2:**
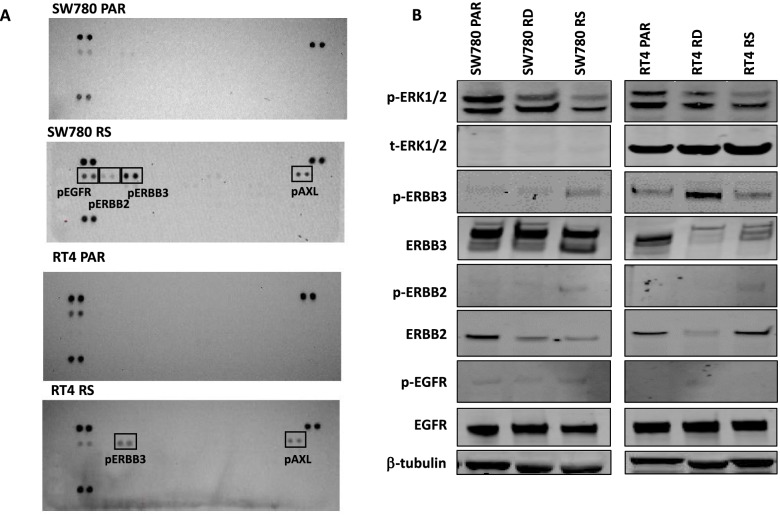
Activation of pERBB receptors in FGFR inhibitor-resistant bladder cancer cell lines. **A** SW780 and RT4 parental (PAR) and Resistant (RS) lines were grown in fresh medium for 24 h without exposure to BGJ398, and cell lysates hybridised to phospho-RTK arrays. Hybridisation signals at the corners serve as controls. **B** Western blot analysis confirming the increase in pERBB3 in SW780 and RT4 parental (PAR) and FGFR inhibitor-resistant (RS) cell lines. Data shown are from a representative experiment

To investigate the mechanistic basis for acquired resistance to FGFR inhibition, the phosphorylation status of 49 receptor tyrosine kinases (RTKs) was compared between parental and SW780-RS and RT4-RS cells using phospho-RTK arrays. This screen identified an increase in pERBB3 in both SW780-RS and RT4-RS cell lines (Fig. 2A), with minimal changes in pEGFR and pERBB2. The increase in pERBB3 in SW780 RS and RT4 RS cells was confirmed by immunoblot, which was also observed in SW780 RD and RT4 RD cells (Fig. 2B). While pAXL levels were also increased in resistant lines, we elected to focus on the increase in pERBB3 in subsequent experiments due to the availability of effective ERBB3 inhibitors.

### Investigation of combination therapy targeting FGFR3 and ERBB3 in FGFR-resistant bladder cancer lines

**Fig. 3:**
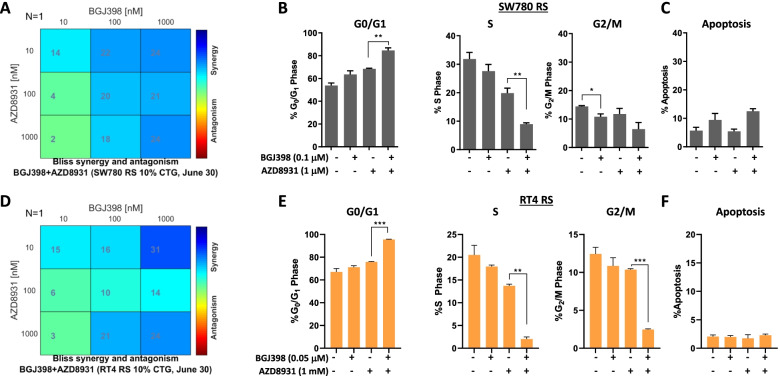
Effect of combinatorial treatment with an FGFR and pan-ERBB inhibitor on (**A, D**) cell viability and (**B**, **E**) cell cycle kinetics, and (**C**, **F**) apoptosis in bladder cancer cell lines with acquired resistance to FGFR inhibitors. **A**, **D** FGFR-inhibitor resistant (**A**) SW780-RS and (**D**) RT4-RS cell lines were treated with a range of concentrations of BGJ398 alone and in combination with the pan-ERBB inhibitor, AZD8931, for 72 h and cell viability assessed using the Cell-Titer Glo assay. Plots shown are the BLISS synergy analysis, which shows synergistic growth inhibition across a range of concentrations. **B**, **E** FGFR inhibitor-resistant (**B**) SW780-RS and (**E**) RT4-RS cell lines were treated with BGJ398 alone and in combination with the pan-ERBB inhibitor, AZD8931, for 24 h and changes in cell cycle distribution determined by propidium iodide staining and FACS analysis. **C**, **F** Assessment of the effect of combination treatment with BGJ398 and AZD8931 on apoptosis by propidium iodide staining and FACS analysis in the same samples analysed in panels **B** and **D**. Values shown are mean ± SEM of a representative experiment performed in triplicate. *P < 0.05 and ***P < 0.0005, t test

Based on the consistent increase in pERBB3 across the resistant lines, we examined the effect of combining BGJ398 with the pan-ERBB family inhibitor AZD8931 in FGFR inhibitor-resistant cell lines [32]. Combination treatment of SW780-RS and RT4-RS cells with BGJ398 and AZD8391 synergistically inhibited cell growth in both SW780-RS and RT4-RS cells (Fig. 3A, D). Assessment of the effect of this combination on cell cycle kinetics revealed a significant reduction in the percentage of cells in S phase and a concomitant increase in the percentage of cells in G0/G1, even when tenfold (SW780-RS) or 20-fold (RT4 RS) lower concentrations of BGJ398 were used compared to the 1 µM dose used to generate resistance (Fig. 3B, E). Comparatively, minimal induction of apoptosis was observed, indicating this combination predominantly induces G0/G1 cell cycle arrest (Fig. 3C, F).

### Reactivation of pERBB3 and MAPK signalling is an early adaptive mechanism of FGFR inhibition

**Fig. 4:**
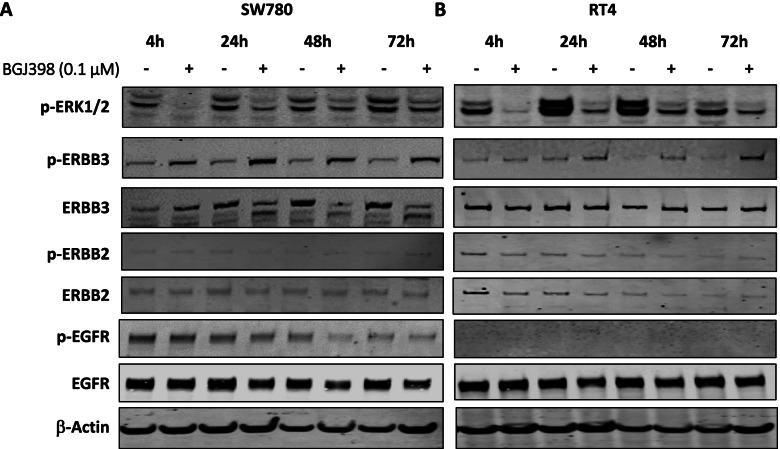
Effect of short-term treatment with BGJ398 on pERK and ERBB family receptors. **A** SW780 and (**B**) RT4 parental cells were treated with BGJ398 for 4–72 h and changes in pERK and ERBB family receptors was determined by western blot. Data shown are from a representative experiment

While these analyses identified mechanisms of acquired resistance associated with long-term FGFR inhibition, it is now evident that several tumours, including bladder cancers, can also rapidly adapt to targeted therapies and reactivate signalling through various mechanisms [25, 33, 34]. We therefore assessed the effect of FGFR inhibition on MAPK signalling in SW780 and RT4 parental cells over 72 h. Remarkably, while BGJ398 initially suppressed pERK levels at 4 h, the magnitude of suppression gradually diminished, and pERK levels rebounded to close to basal levels by 72 h (Fig. 4A, B). We next investigated whether this feedback was associated with changes in ERBB3 and other ERBB family members. While minimal induction of pERBB2 or pEGFR was observed over the 72-h time course in either cell line, robust induction of pERBB3 was observed in both cell lines within 24 h.

**Fig. 5:**
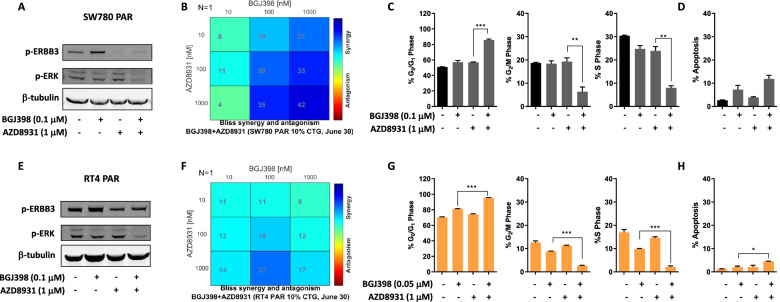
Effect of combinatorial treatment of parental FGFR3 fusion harboring bladder cell lines with an FGFR and pan-ERBB inhibitor on (**A**, **E**) cell signaling, (**B**, **F**) cell viability, (**C**, **G**) cell cycle kinetics and (**D**, **H**) apoptosis. **A**, **E** Parental SW780 and RT4 cells cells were treated with BGJ398 (0.1 µM) or AZD8931 (1 µM), alone or in combination for 72 h and changes in pERBB3 and pERK determined by western blot. **B**, **F** Parental SW780 and RT4 cells were treated with a range of concentrations of BGJ398 or AZD8931 alone or in combination for 72 h and cell viability determined using Cell-Titer Glo assays. Plots shown are the BLISS synergy analysis from a representative experiment, which shows synergistic growth inhibition across a range of concentrations. **C**, **G** FGFR inhibitor-resistant (**C**) SW780-RS and (**G**) RT4-RS cell lines were treated with BGJ398 alone and in combination with the pan-ERBB inhibitor, AZD8931, for 24 h and changes in cell cycle distribution determined by propidium iodide staining and FACS analysis. **D**, **H** Assessment of the effect of combination treatment with BGJ398 and AZD8931 on apoptosis by propidium iodide staining and FACS analysis in the same samples analysed in panels **C** and **G**. Values shown are mean ± SEM of a representative experiment performed in triplicate. *P < 0.05 and ***P < 0.0005, t test

We therefore investigated whether the rapid reactivation of MAPK signalling in FGFR-fusion harbouring bladder cancer cells could be attenuated by combined treatment with an FGFR inhibitor and the pan-ERBB inhibitor, AZD8931, and whether this could enhance the growth inhibitory effect of FGFR inhibitors. Indeed, combined treatment of both RT4 and SW780 parental cells with BGJ398 and AZD8931 significantly attenuated the induction of pERBB3, and further suppressed pERK levels compared to the effect of either agent alone (Fig. 5A, E). Furthermore, combination treatment with BGJ398 and AZD8931 synergistically inhibited cell growth in both SW780 and RT4 cells (Fig. 5B, F). Notably, this effect was observed at a concentration of 0.1 and 0.05 µM BGJ398 in SW780 and RT4 cells respectively, which is 5–tenfold lower than the clinically achievable concentration of 0.5 µM [30]. As observed in the long-term resistance setting, the combination induced a significant reduction in the percentage of cells in S phase and a concomitant increase in the percentage of cells in G0/G1 (Fig. 5C, G). Comparatively, minimal induction of apoptosis was observed, indicating this combination predominantly induces G0/G1 cell cycle arrest (Fig. 5D, H).

**Fig. 6:**
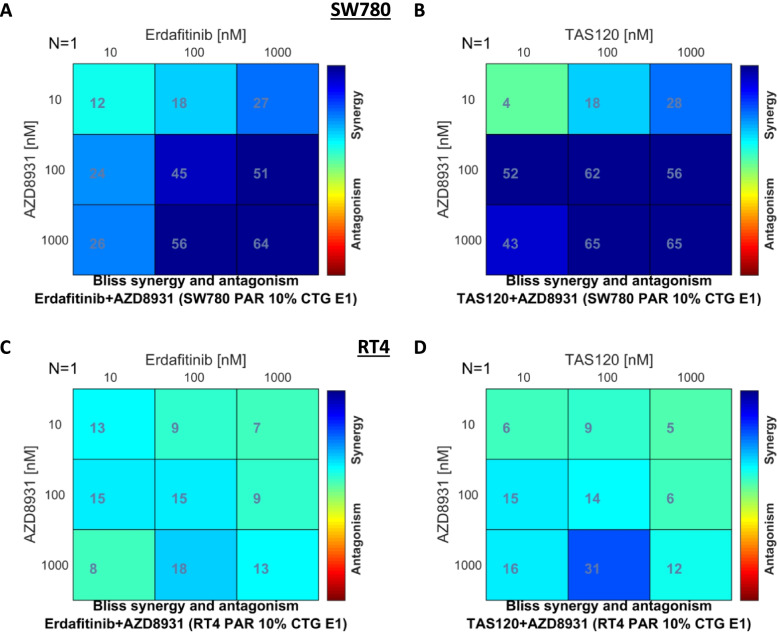
Effect of combinatorial treatment of parental FGFR3 fusion harboring bladder cell lines with (**A**, **C**) erdafitinib and AZD8931, or (**B**, **D**) TAS-120 and AZD8931 on cell viability. **A**, **B** Parental SW780 and (**C, D**) RT4 cells were treated with a range of concentrations of BGJ398 or AZD8931 alone or in combination for 72 h and cell viability determined using Cell-Titer Glo assays. Plots shown are the BLISS synergy analysis from a representative experiment, which shows synergistic growth inhibition across a range of concentrations

Finally, we assessed whether synergistic suppression of cell proliferation of these *FGFR3*-fusion harboring cell lines was also induced when the FGFR inhibitors erdafitinib and TAS-120 were combined with AZD8931. Indeed, combining either erdafitinib or TAS-120 with AZD8931 induced synergistic suppression of proliferation of SW780 and RT4 parental cells, demonstrating this is a highly effective combination regimen that can enhance the activity of multiple FGFR inhibitors (Fig. 6A-D).

## Discussion

FGFR inhibitors such as BGJ398 and the clinically approved agent erdafitinib induce objective responses in ~ 25–40% of patients with metastatic urothelial cancer harbouring *FGFR3* alterations [23, 24, 35]. However, the median progression-free survival in these patients is typically less than six months. These findings point to the existence of two types of resistance – inherent resistance which precludes 60–70% of patients responding at all, and acquired resistance, where tumours which initially respond invariably develop resistance. These observations are similar to the extensive clinical experience with other targeted therapies where initial responses are often followed by tumour progression after 6–12 months [36–38]. To better understand the mechanisms of inherent and acquired resistance to FGFR inhibition, we undertook *in vitro* modelling of these processes in two previously uncharacterised *FGFR3*-fusion driven cell lines, SW780 (*FGFR3-BAIP2L1* fusion) and RT4 (*FGFR3-TACC3* fusion).

Interestingly, a prior study reported that not all *FGFR3* altered cell lines remain dependent on FGFR for growth *in vitro*, with some cell lines switching their dependence to ERBB family members [26]. While the basis for this switch is unknown, it may reflect differences in the availability of ERBB family ligands in the serum, as the exogenous provision of the ERBB2/3 ligands NRG1 or NRG2 has been shown to attenuate the sensitivity of FGFR3-fusion harboring RT-112 cells to BGJ398 [25]. Notably, while both of the *FGFR3*-fusion harbouring cell lines investigated in the current study were sensitive to FGFR inhibition, we did note considerable variability in response in the SW780 cell line with different serum batches and serum concentrations (data not shown), indicating this is an important consideration in these studies.

In this study, we examined resistance mechanisms to FGFR inhibition in two contexts – acquired resistance following long term exposure, and rapidly following 4–72 h treatment – in two bladder cancer cell lines harbouring *FGFR3*-fusions that had not been previously characterized in terms of response to FGFR inhibition. While profiling of changes in receptor tyrosine kinases in cell lines with acquired resistance identified increased expression of several candidates, only expression of pERBB3 was consistently increased in both cell line models and was independent of the mode of resistance induction (RS vs RD). Importantly, combination treatment with the pan-ERBB inhibitor AZD8931 profoundly re-sensitised these lines to FGFR inhibition, indicating a direct role for pERBB3 in conferring resistance. This finding is consistent with observations previously reported in RT-112 cells [25, 39], suggesting this may be a common resistance mechanism in these tumours.

An important finding of the current study is the capacity of *FGFR3*-fusion harbouring bladder cancer cells to rapidly reactivate MAPK signalling. We demonstrated that this is associated with increased pERBB3 levels, and that the increase in pERBB3 and reactivation of pERK could be attenuated by combined treatment with the pan-ERBB inhibitor, AZD8931. In parallel, combined FGFR/ERBB inhibition further inhibited cell growth of FGFR3-fusion driven bladder cancer lines. These findings are consistent with those previously reported in RT-112 cells, where pERBB2 and pERBB3 were both rapidly elevated within 24 h of BGJ398 treatment [25]. Furthermore, Herrera-Abreu et al. also demonstrated that RT-112 cells express high basal levels of EGFR, and that EGFR-signaling is rapidly induced following BGJ398 treatment, limiting its response to BGJ398 [26]. While we did not observe a rapid increase in pERBB2 or pEGFR in SW780 and RT4 cells following BGJ398 treatment, both receptors are expressed in these cells, particularly EGFR which is expressed at high levels. As ERBB3 can activate signalling through hetero-dimerisation with ERBB2 and EGFR [40], it is possible that ERBB3 may heterodimerise with existing ERBB2 and EGFR to reactivate MAPK signalling in these cells. On the other hand, cross-talk between ERBB and FGFR receptors has also been described, with ERBB3 required for the maintenance of FGFR2 phosphorylation and proliferation in some *FGFR2*-amplified gastric cancer cells [41]. A further possibility therefore is that ERBB3 may cooperate with FGFR3 to reactivate MAPK/ERK signalling. While additional studies are required to define the specific mechanisms by which ERBB receptors contribute to signalling in *FGFR3*-fusion driven bladder cancers following FGFR inhibition, our findings in the SW780 and RT4 cell lines adds to the previous findings in RT-112 cells to demonstrate compensatory activation of ERBB3-driven signalling as a consistent mechanism of rapid adaptive resistance to FGFR inhibitors in these tumours.

Critical to preventing the emergence of resistance is the more efficient targeting and elimination of FGFR-driven tumours from the onset of treatment. In this regard, the finding that compensatory activation of ERBB3-driven signalling occurs rapidly in response to FGFR inhibition in multiple models of *FGFR3*-fusion harbouring bladder cancer, and our demonstration that dual targeting of FGFR and ERBB family receptors synergistically inhibits growth, suggests that dual blockade of FGFR and ERBB-driven signalling may represent a more effective treatment strategy for these tumours. Additionally, ERBB3-targeting antibodies are now entering clinical trial for other indications (e.g. seribantumab for NRG1-fusion harboring tumours) [42] (NCT04383210), which may provide further options for clinical investigation of this treatment concept.

Finally, while these studies establish a role of increased ERBB3 phosphorylation in driving resistance to FGFR inhibition, additional studies are needed to understand the specific mechanisms driving this event. Possibilities include the upregulation of ERBB3 ligands [25] which can be assessed by gene expression profiling or ELISA-based approaches. Finally, it is possible that other mechanisms may also be involved in driving resistance to FGFR inhibition. Comprehensive genomic and transcriptomic profiling of the resistant cell lines generated in this study could unveil such mechanisms and provide further opportunities for enhancing the efficacy of these treatments.

## Conclusions

In summary, we identify consistent increased activation of pERBB3 as a resistance mechanism to FGFR inhibition in *FGFR3*-fusion driven bladder cancer cell lines. Our findings suggest that upfront combination treatment with FGFR and ERBB3 inhibitor warrants further investigation for *FGFR3*-fusion driven bladder cancers.

## Supplementary Information

**Additional file 1.**

## Data Availability

All data generated or analysed during this study are included in this published article [and its supplementary information files].
