# Supplementary material for: Dual targeting of FGFR3 and ERBB3 enhances the efficacy of FGFR inhibitors in FGFR3 fusion-driven bladder cancer
Source: BMC Cancer. 2022 May 2;22:478. doi: 10.1186/s12885-022-09478-4 (PMC9063072; doi:10.1186/s12885-022-09478-4)

Supplementary Table 1: Short tandem repeat profiling of SW780 and RT4 parental and resistant cell lines.

Sample Name	Marker	Allele 1	Allele 2	Size 1	Size 2
SW780	AMEL	X	X	103.97	103.97
SW780_PAR	AMEL	X	X	104.07	104.07
SW780_RD	AMEL	X	X	104.05	104.05
SW780_RS	AMEL	X	X	104.07	104.07
RT4	AMEL	X	Y	104.02	109.82
RT4_PAR	AMEL	X	Y	104.09	109.86
RT4_RD	AMEL	X	Y	104.02	109.82
RT4_RS	AMEL	X	Y	104.09	109.86
SW780	CSF1PO	10	11	333.47	337.5
SW780_PAR	CSF1PO	10	11	333.56	337.59
SW780_RD	CSF1PO	10	11	333.53	337.55
SW780_RS	CSF1PO	10	11	333.52	337.54
RT4	CSF1PO	10	12	333.55	341.61
RT4_PAR	CSF1PO	10	12	333.55	341.61
RT4_RD	CSF1PO	10	12	333.5	341.58
RT4_RS	CSF1PO	10	12	333.55	341.61
SW780	D13S317	11	12	187.85	191.76
SW780_PAR	D13S317	11	12	187.91	191.72
SW780_RD	D13S317	11	12	187.9	191.71
SW780_RS	D13S317	11	12	187.9	191.8
RT4	D13S317	8	8	175.88	175.88
RT4_PAR	D13S317	8	8	175.94	175.94
RT4_RD	D13S317	8	8	175.86	175.86
RT4_RS	D13S317	8	8	175.97	175.97
SW780	D16S539	9	11	278.86	286.88
SW780_PAR	D16S539	9	11	278.84	286.84
SW780_RD	D16S539	9	11	278.76	286.84
SW780_RS	D16S539	9	11	278.83	286.89
RT4	D16S539	9	9	278.81	278.81
RT4_PAR	D16S539	9	9	278.81	278.81
RT4_RD	D16S539	9	9	278.83	278.83
RT4_RS	D16S539	9	9	278.8	278.8
SW780	D21S11	29	30	219.42	223.48
SW780_PAR	D21S11	29	30	219.35	223.4
SW780_RD	D21S11	29	30	219.44	223.48
SW780_RS	D21S11	29	30	219.46	223.4
RT4	D21S11	30	32.2	223.38	233.41
RT4_PAR	D21S11	30	32.2	223.47	233.5
RT4_RD	D21S11	30	32.2	223.38	233.43
RT4_RS	D21S11	30	32.2	223.38	233.41
SW780	D5S818	11	12	130.61	134.83
SW780_PAR	D5S818	11	12	130.66	134.86
SW780_RD	D5S818	11	12	130.56	134.76
SW780_RS	D5S818	11	12	130.61	134.79
RT4	D5S818	11	12	130.62	134.74
RT4_PAR	D5S818	11	12	130.57	134.81
RT4_RD	D5S818	11	12	130.57	134.81
RT4_RS	D5S818	11	12	130.57	134.8
SW780	D7S820	9	10	224.24	228.29
SW780_PAR	D7S820	9	10	224.16	228.29
SW780_RD	D7S820	9	10	224.24	228.28
SW780_RS	D7S820	9	10	224.16	228.28
RT4	D7S820	9	12	224.32	236.29
RT4_PAR	D7S820	9	12	224.32	236.38
RT4_RD	D7S820	9	12	224.23	236.33
RT4_RS	D7S820	9	12	224.32	236.38
SW780	TH01	6	6	161.91	161.91
SW780_PAR	TH01	6	6	161.91	161.91
SW780_RD	TH01	6	6	161.99	161.99
SW780_RS	TH01	6	6	161.99	161.99
RT4	TH01	9	9.3	173.81	176.74
RT4_PAR	TH01	9	9.3	173.87	176.81
RT4_RD	TH01	9	9.3	173.78	176.72
RT4_RS	TH01	9	9.3	173.9	176.83
SW780	TPOX	8	8	268.93	268.93
SW780_PAR	TPOX	8	8	268.93	268.93
SW780_RD	TPOX	8	8	268.89	268.89
SW780_RS	TPOX	8	8	268.95	268.95
RT4	TPOX	8	11	268.89	280.88
RT4_PAR	TPOX	8	11	268.91	280.88
RT4_RD	TPOX	8	11	268.87	280.9
RT4_RS	TPOX	8	11	268.89	280.86
SW780	vWA	16	19	146.24	158.16
SW780_PAR	vWA	16	19	146.21	158.17
SW780_RD	vWA	16	19	146.24	158.25
SW780_RS	vWA	16	19	146.24	158.25
RT4	vWA	14	17	138.31	150.23
RT4_PAR	vWA	14	17	138.3	150.18
RT4_RD	vWA	14	17	138.3	150.23
RT4_RS	vWA	14	17	138.3	150.23

Full length western blots

Fig 2b

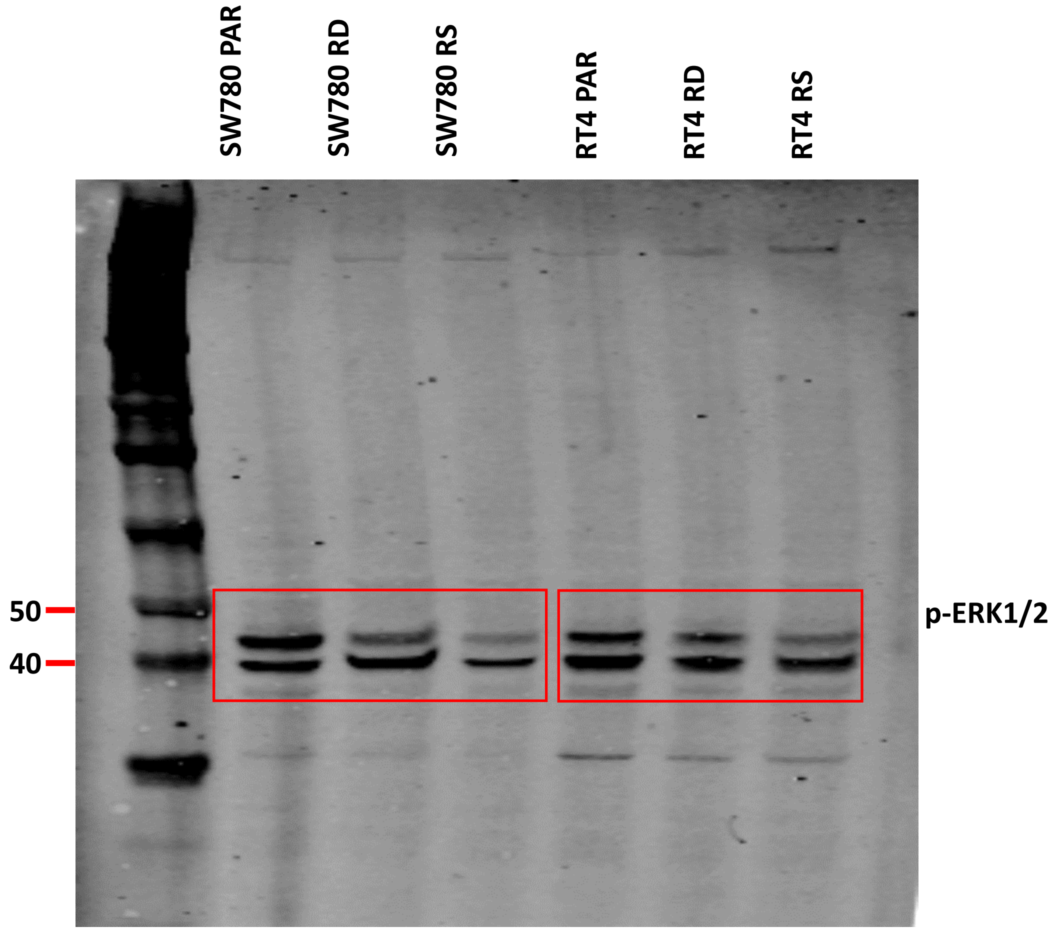

* This image shown represents the full western blot. All edges not present due to the scanned image border being smaller than the western membrane.

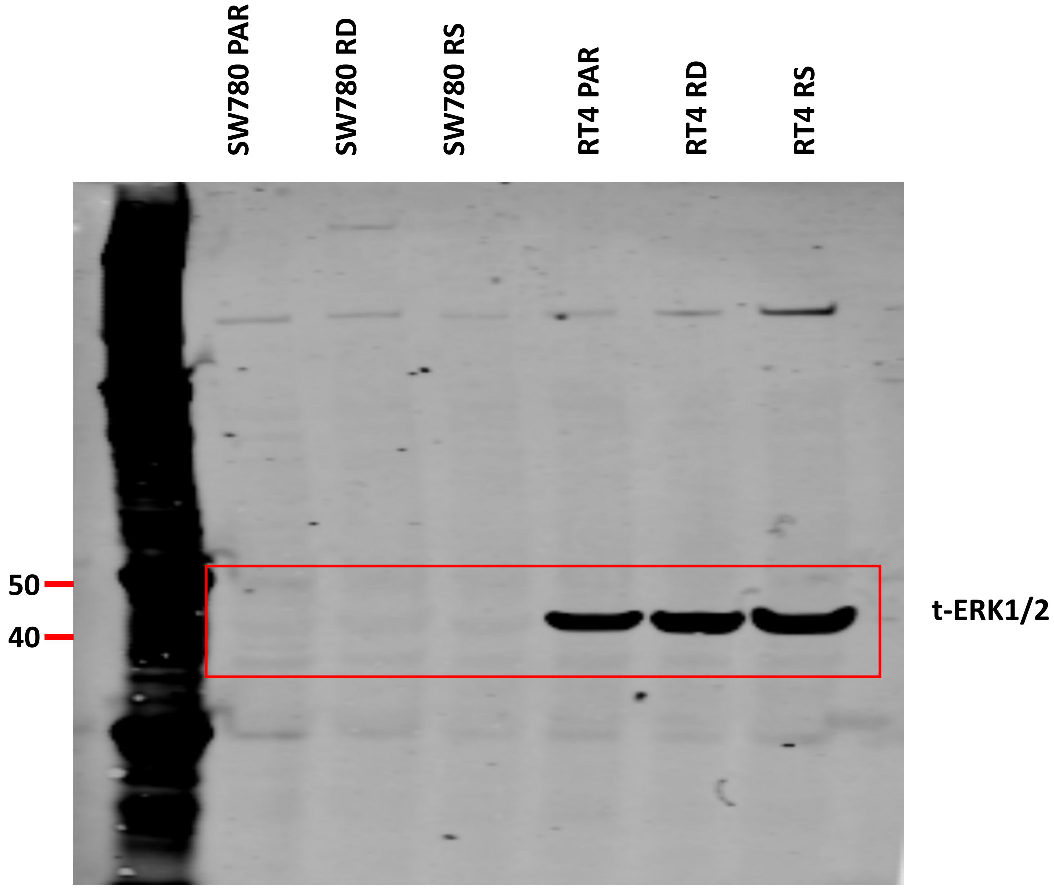

* This image shown represents the full western blot. Edges not present due to the scanned image border being smaller than the western membrane.

Fig 2b

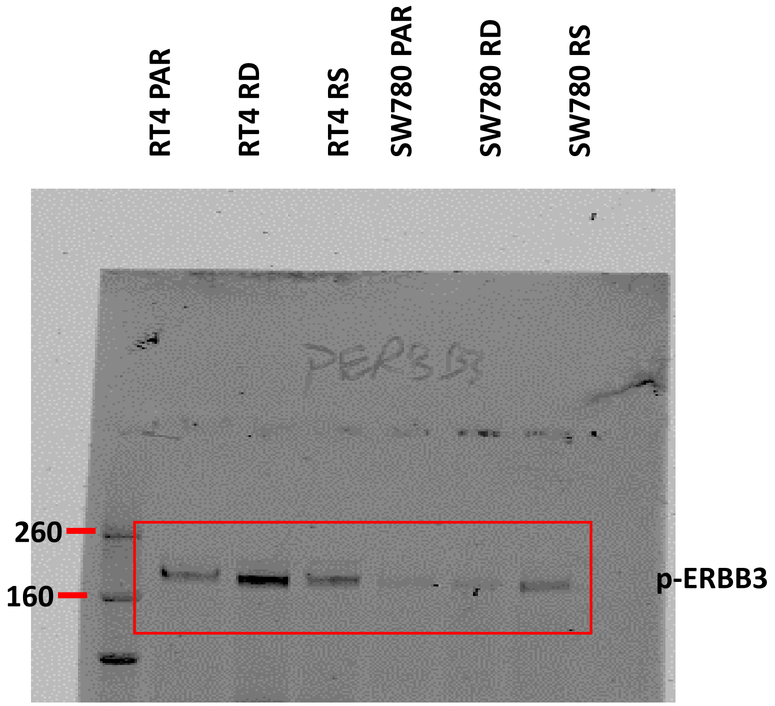

RT4 PAR
RT4 RD
RT4 RS
SW780 PAR
SW780 RD
SW780 RS
260
160
p-ERBB3

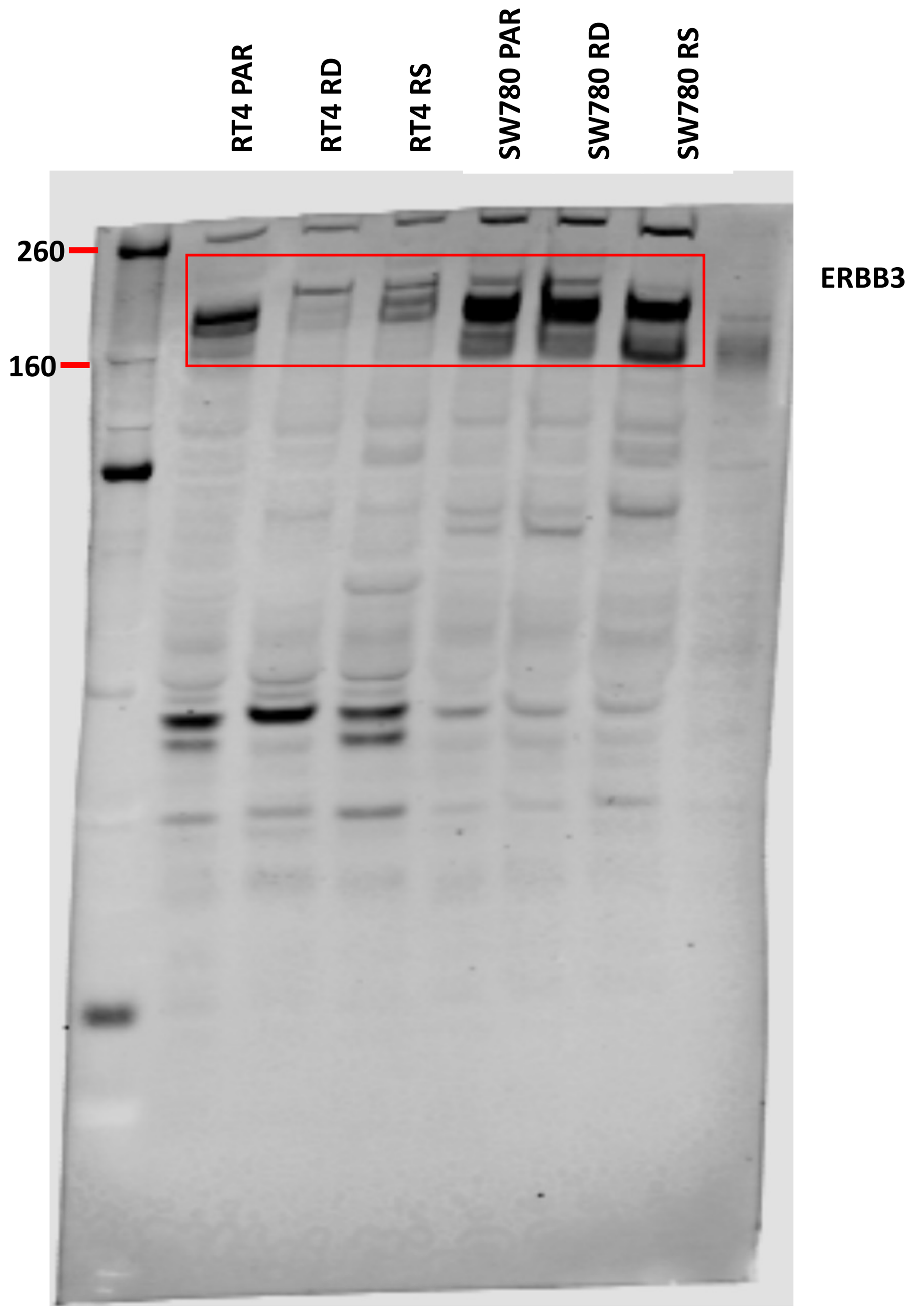

RT4 PAR
RT4 RD
RT4 RS
SW780 PAR
SW780 RD
SW780 RS
260
160
ERBB3

Fig 2b

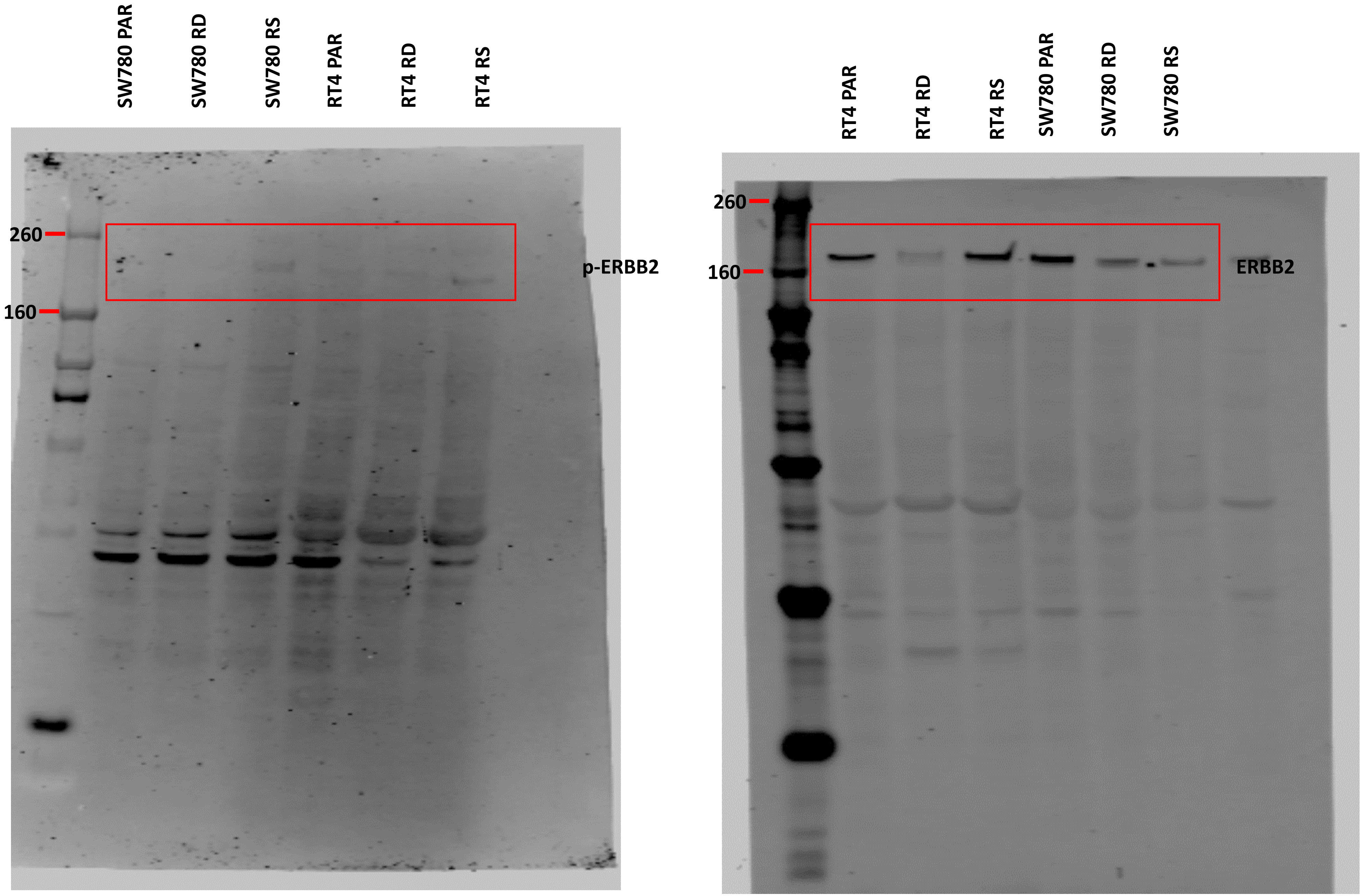

SW780 PAR
SW780 RD
SW780 RS
RT4 PAR
RT4 RD
RT4 RS
260
160
p-ERBB2
RT4 PAR
RT4 RD
RT4 RS
SW780 PAR
SW780 RD
SW780 RS
260
160
ERBB2

Fig 2b

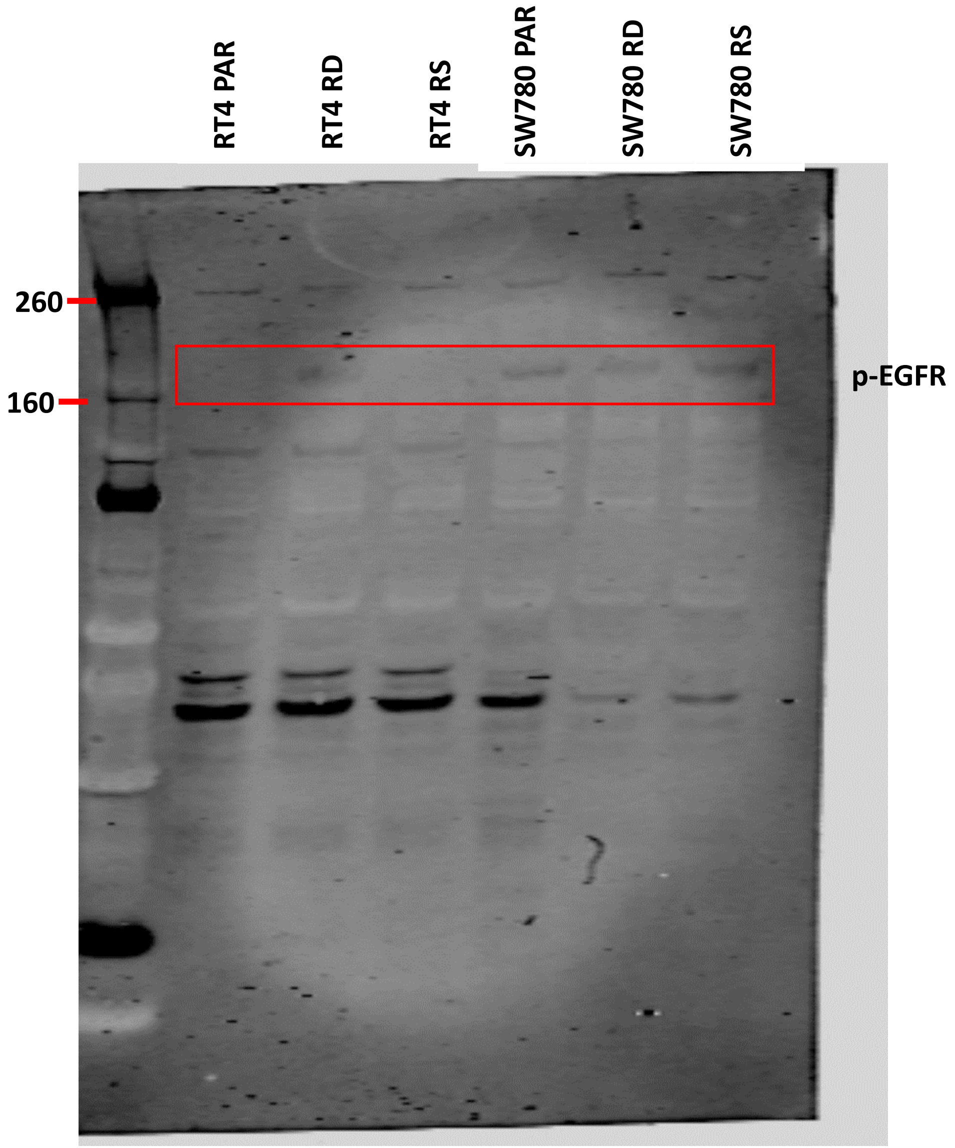

RT4 PAR
RT4 RD
RT4 RS
SW780 PAR
SW780 RD
SW780 RS
260
160
p-EGFR

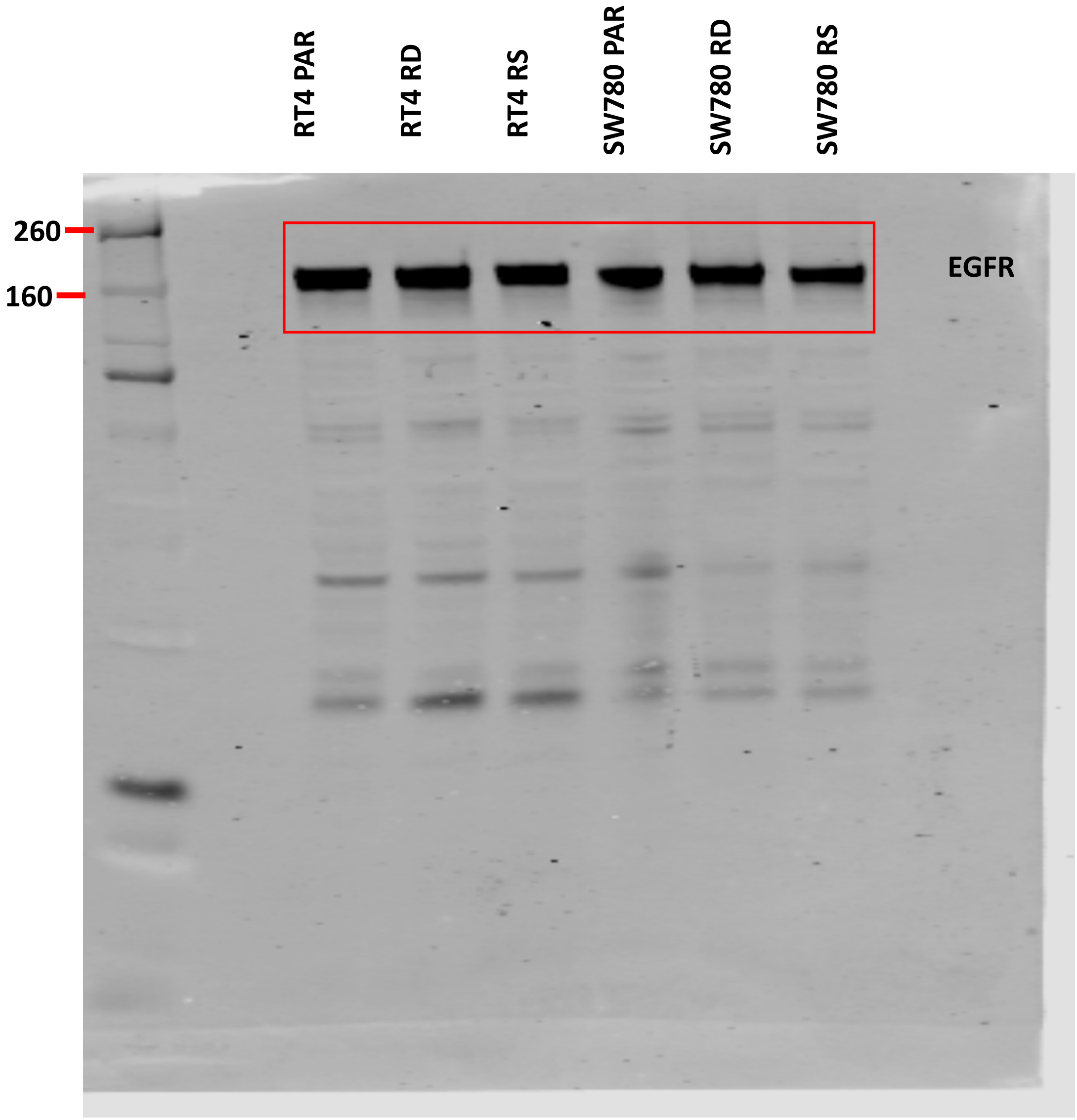

RT4 PAR
RT4 RD
RT4 RS
SW780 PAR
SW780 RD
SW780 RS
260
160
EGFR

Fig 2b

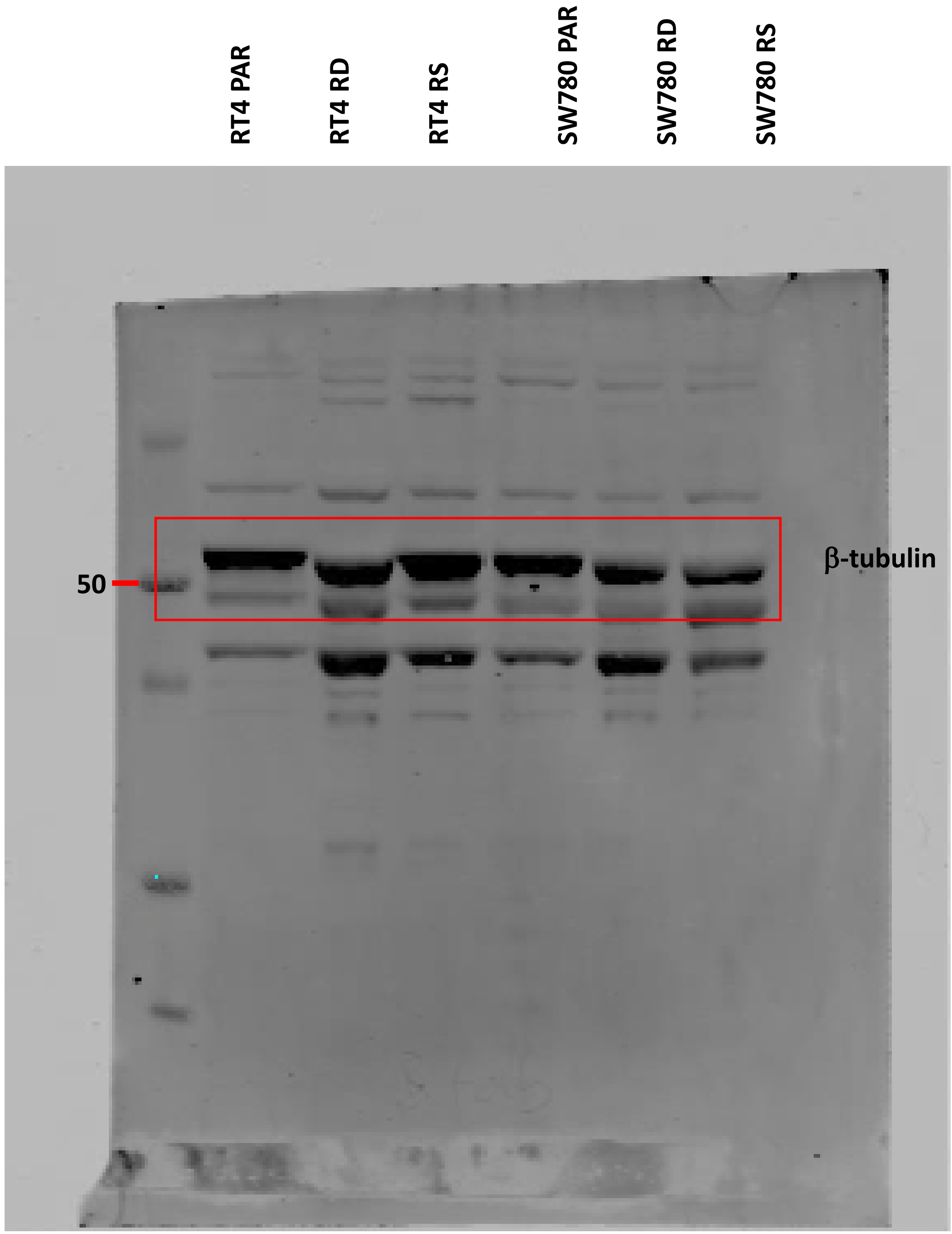

Fig 4

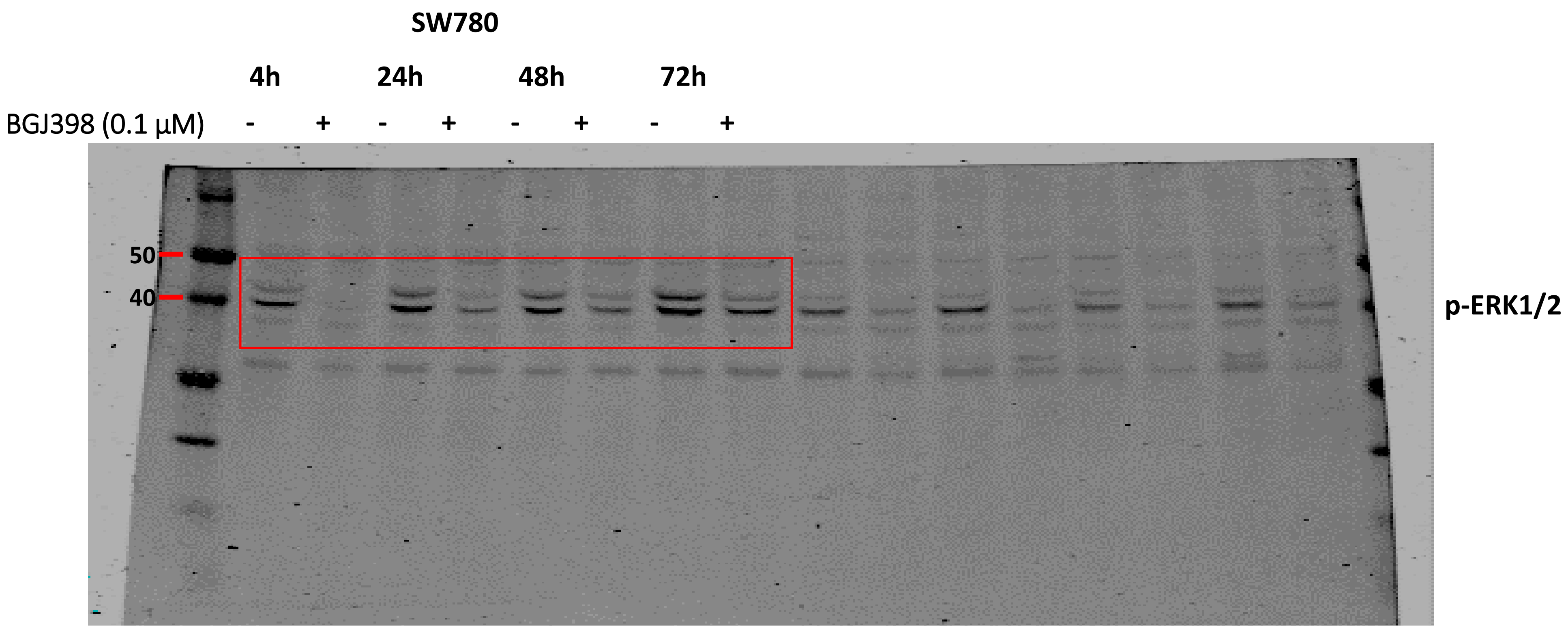

Fig 4

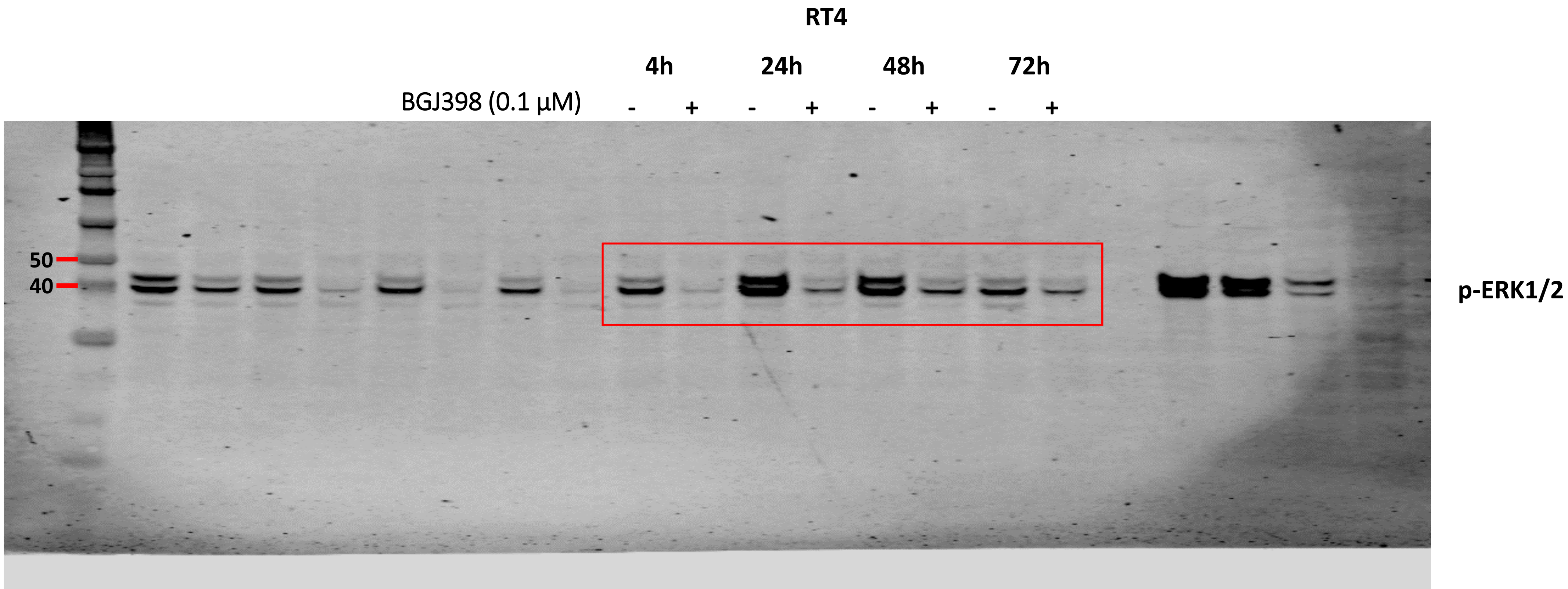

Fig 4

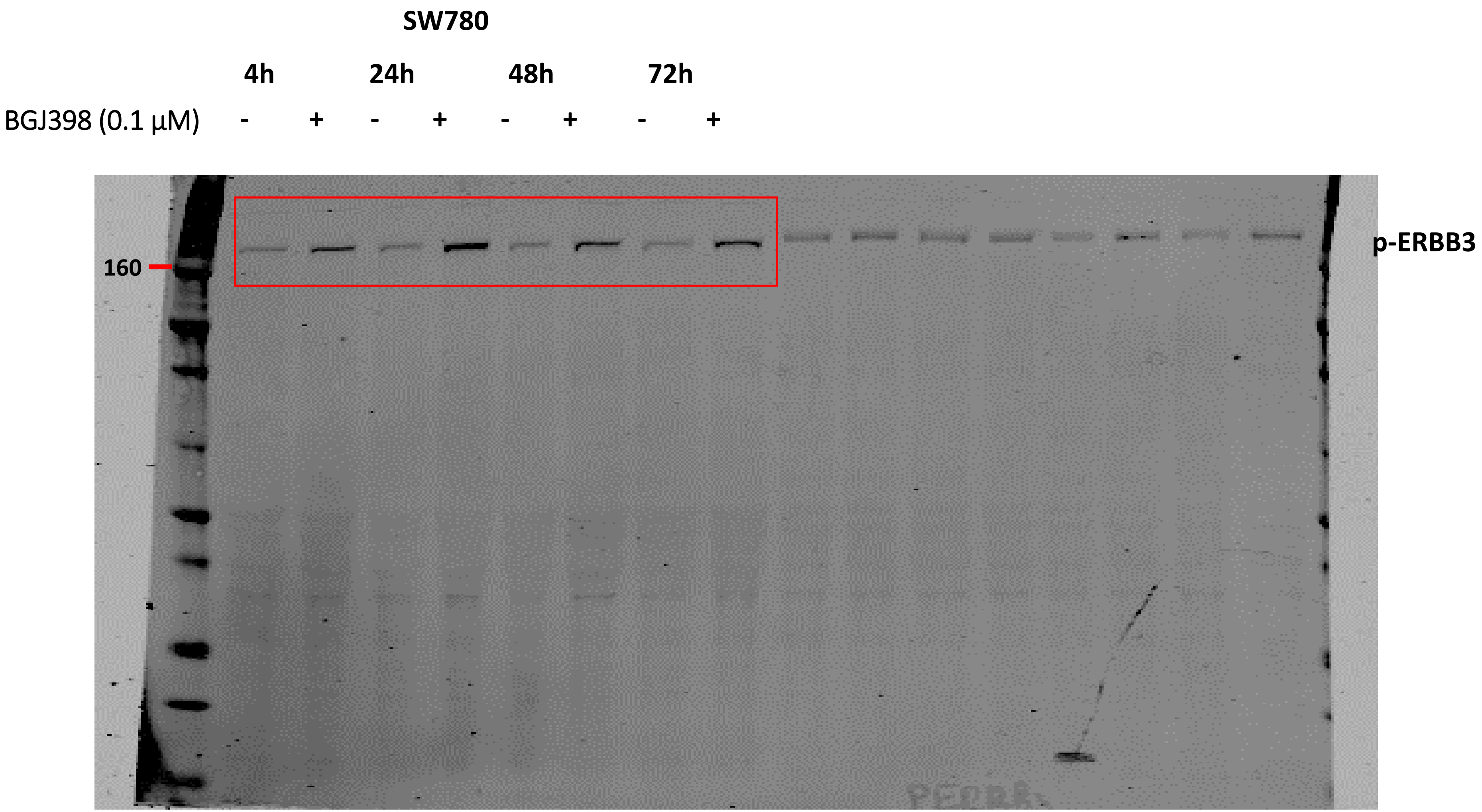

Fig 4

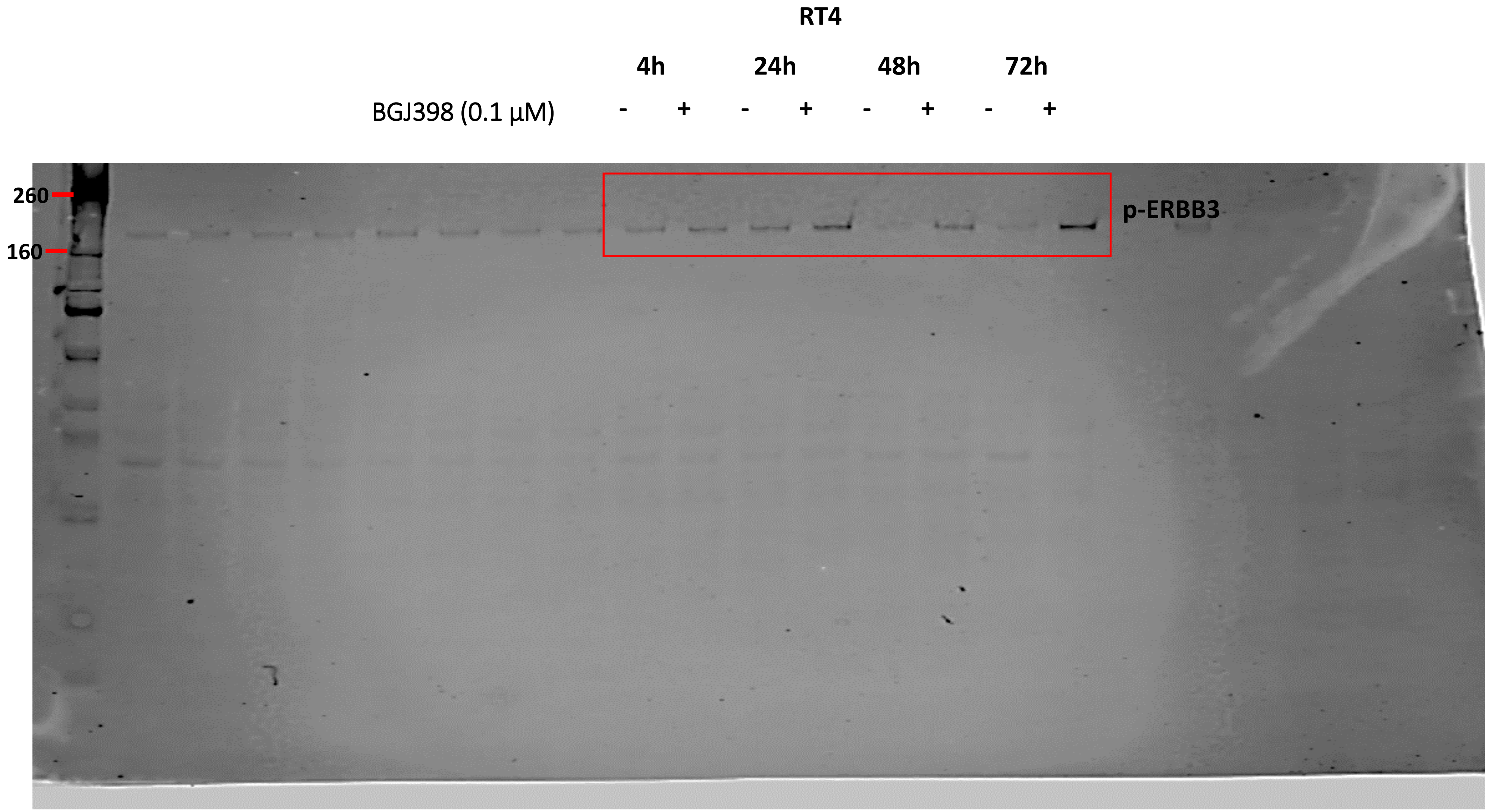

Fig 4

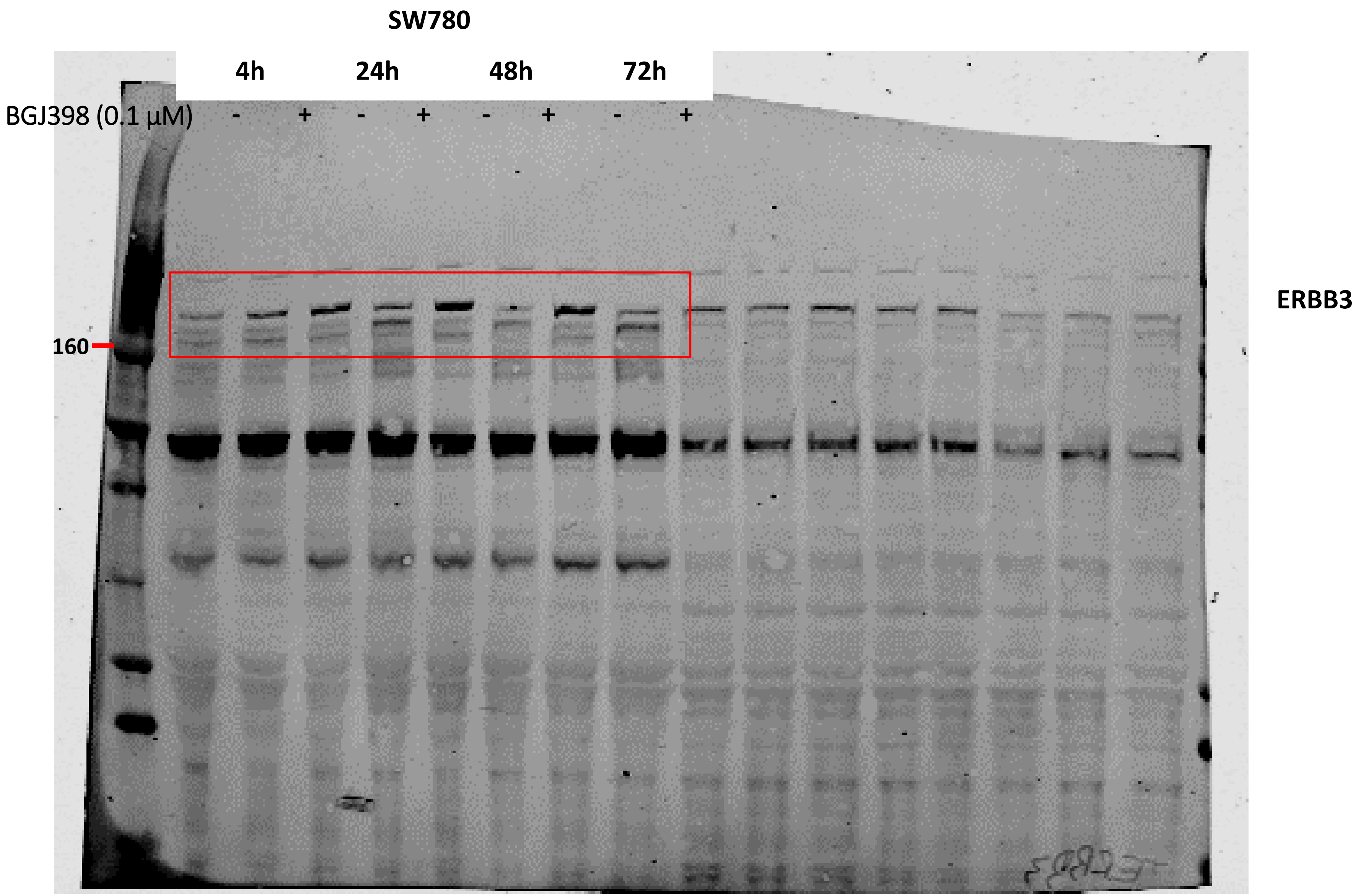

Fig 4

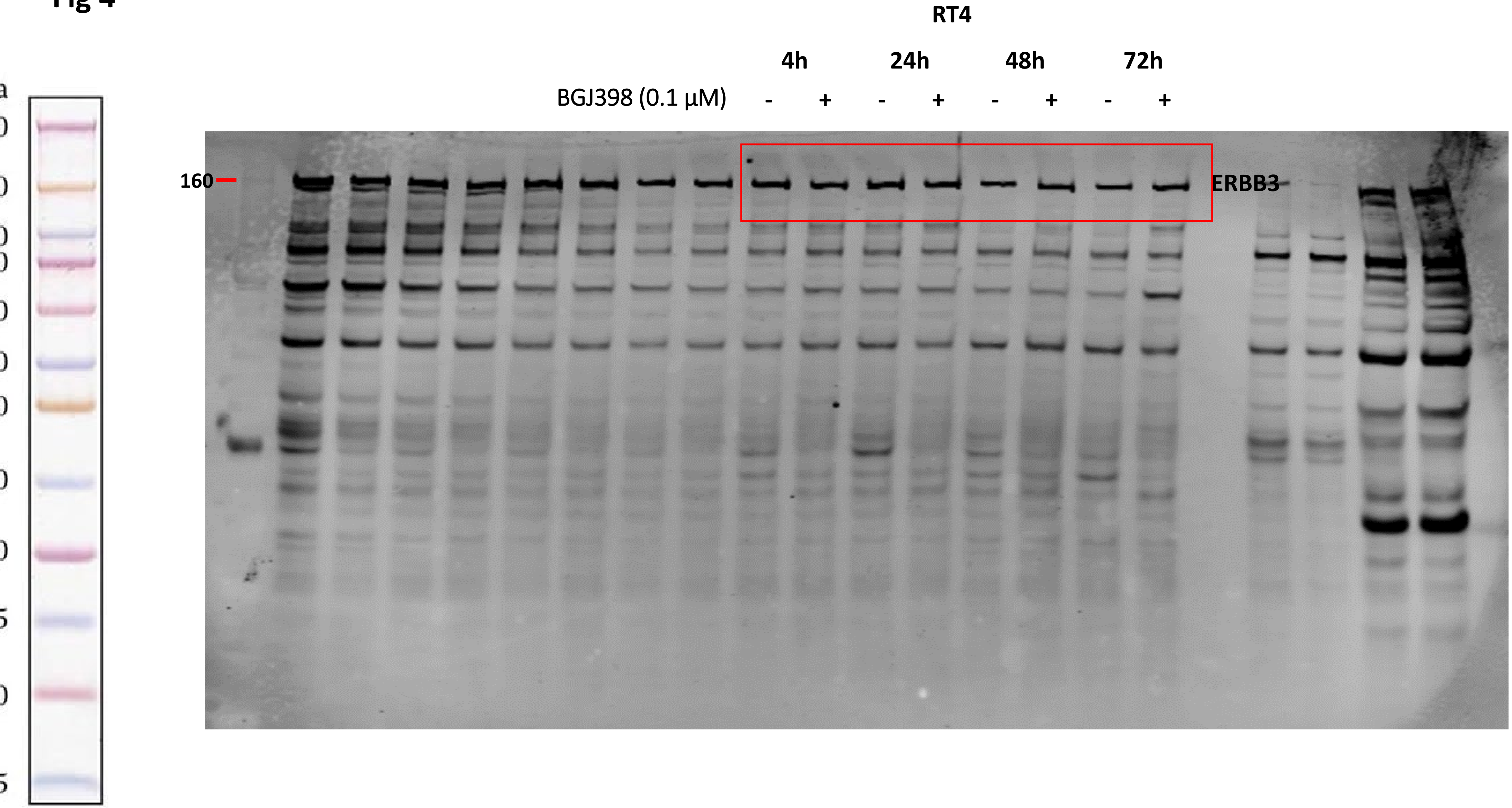

* This image shown represents the full western blot. Edges were not present due to the scanned image border being smaller than the western membrane.

Fig 4

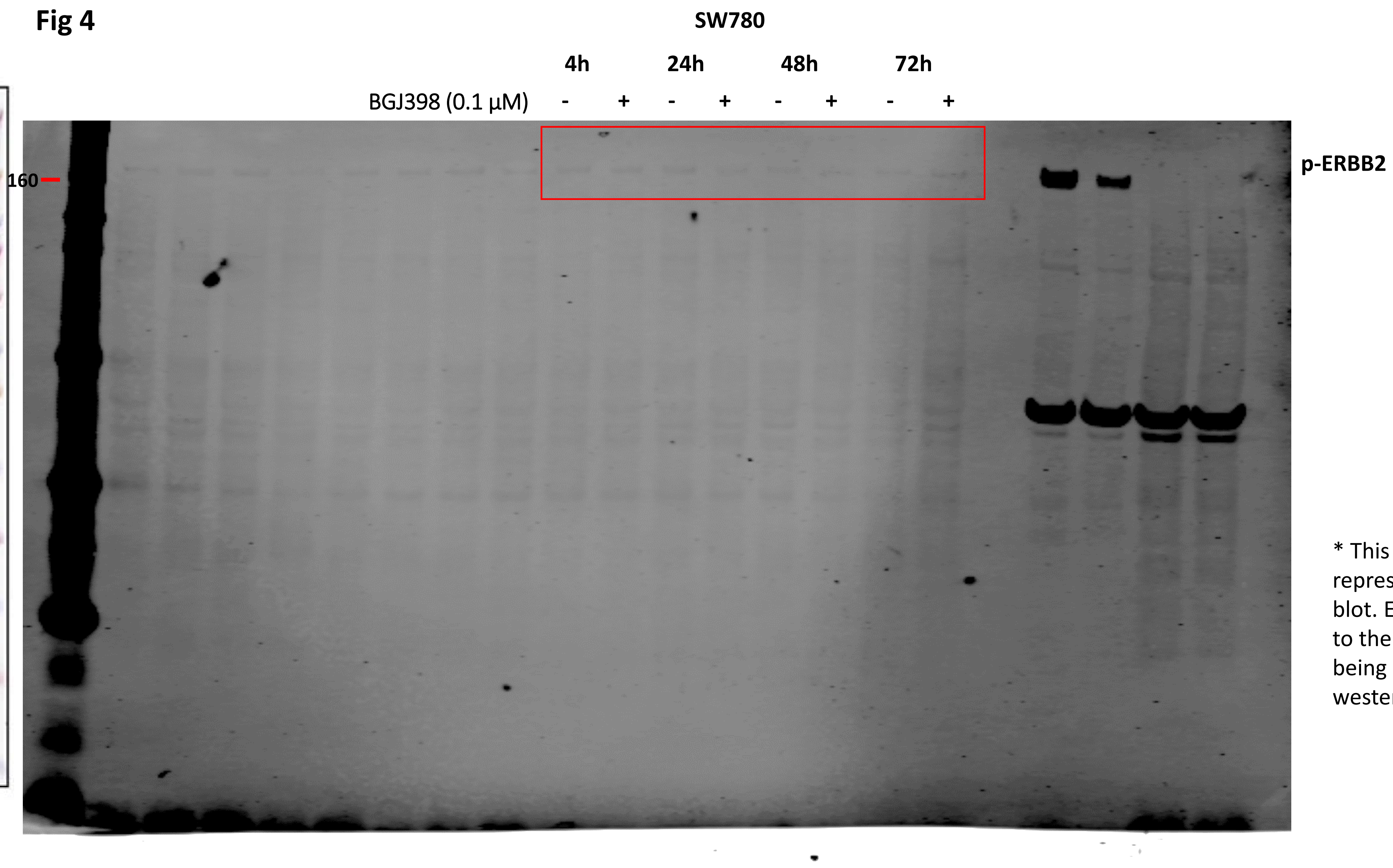

* This image shown represents the full western blot. Edges not present due to the scanned image border being smaller than the western membrane.

Fig 4

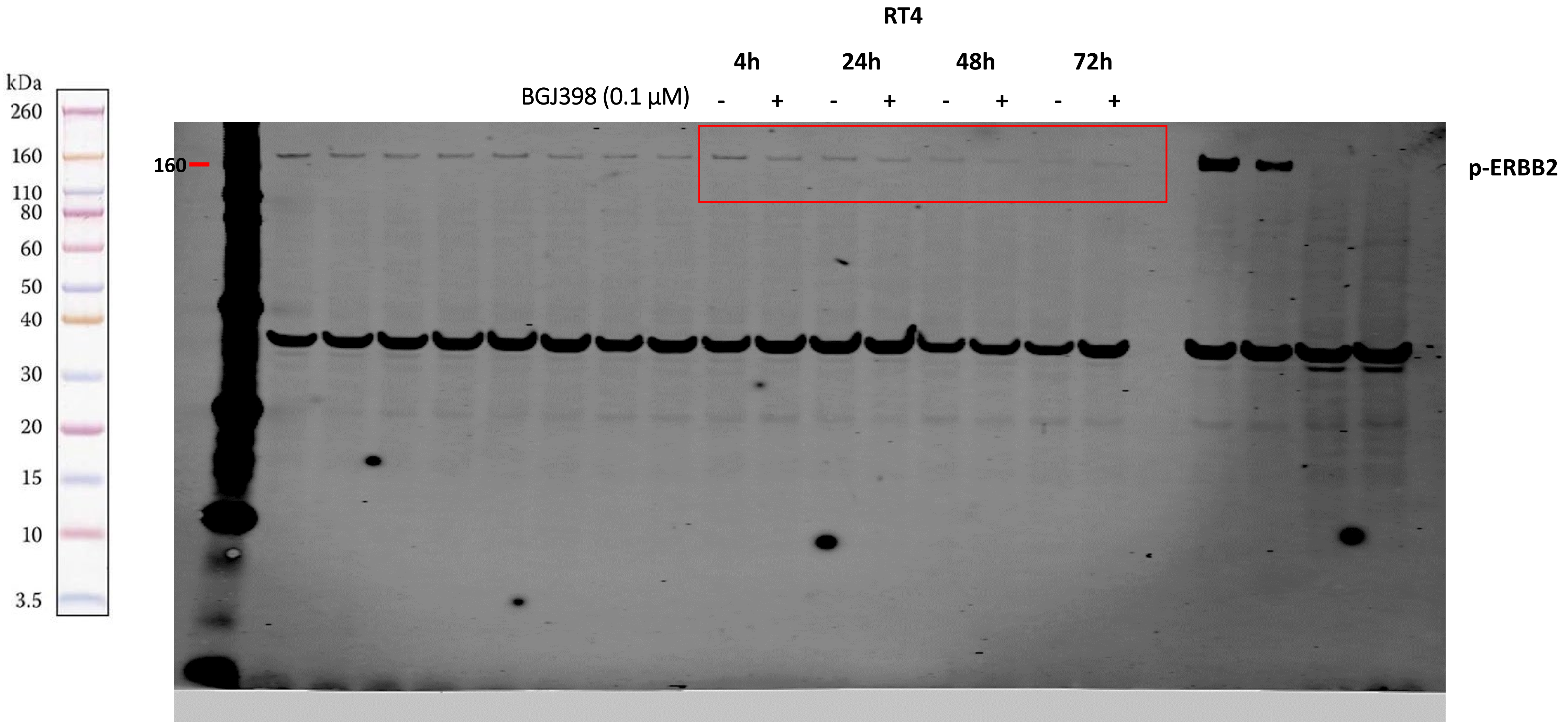

kDa
260
160
110
80
60
50
40
30
20
15
10
3.5
RT4
4h
24h
48h
72h
BGJ398 (0.1 µM)
-
+
-
+
-
+
-
+
160
p-ERBB2

Fig 4

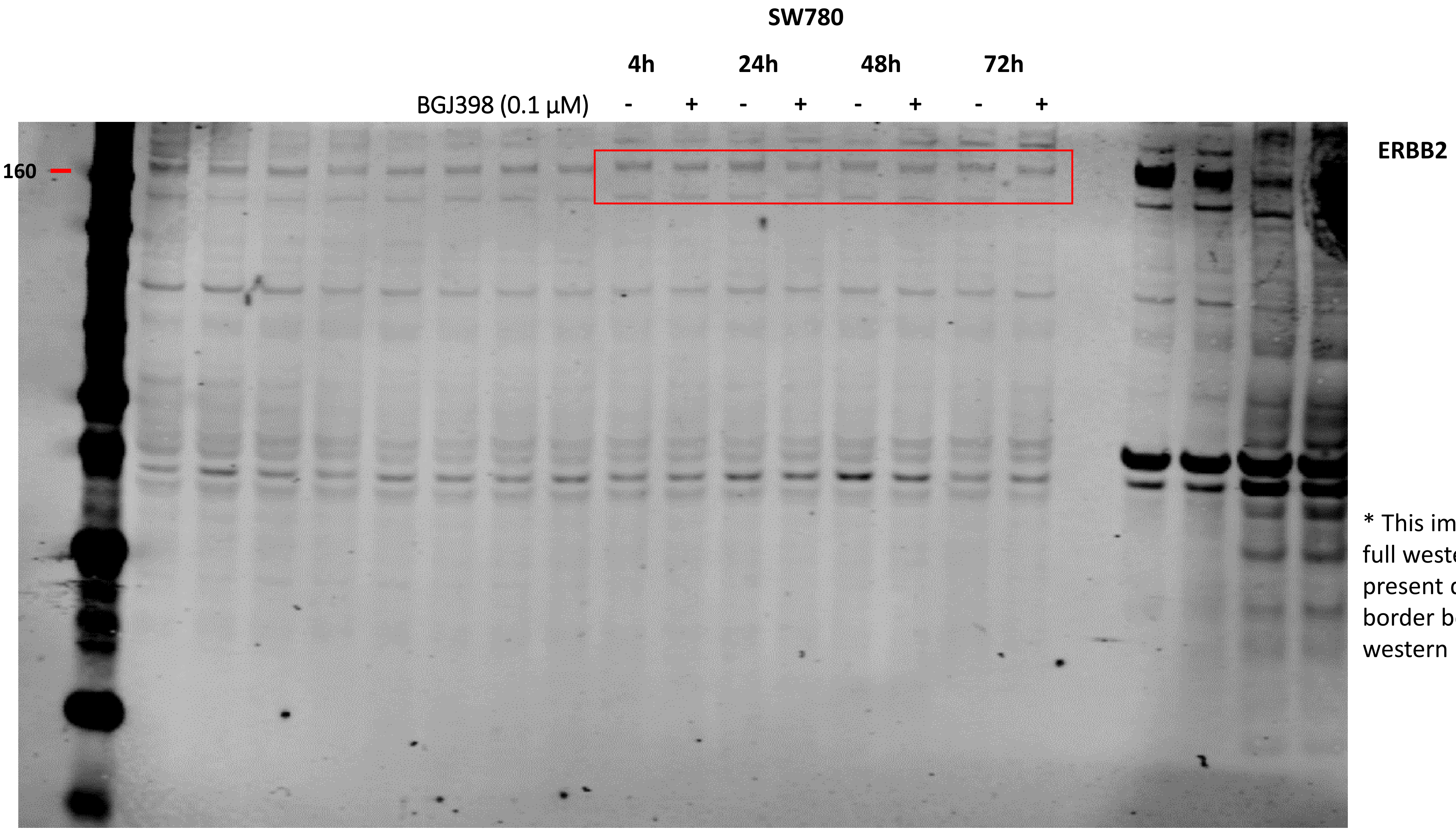

* This image shown represents the full western blot. Edges were not present due to the scanned image border being smaller than the western membrane.

Fig 4

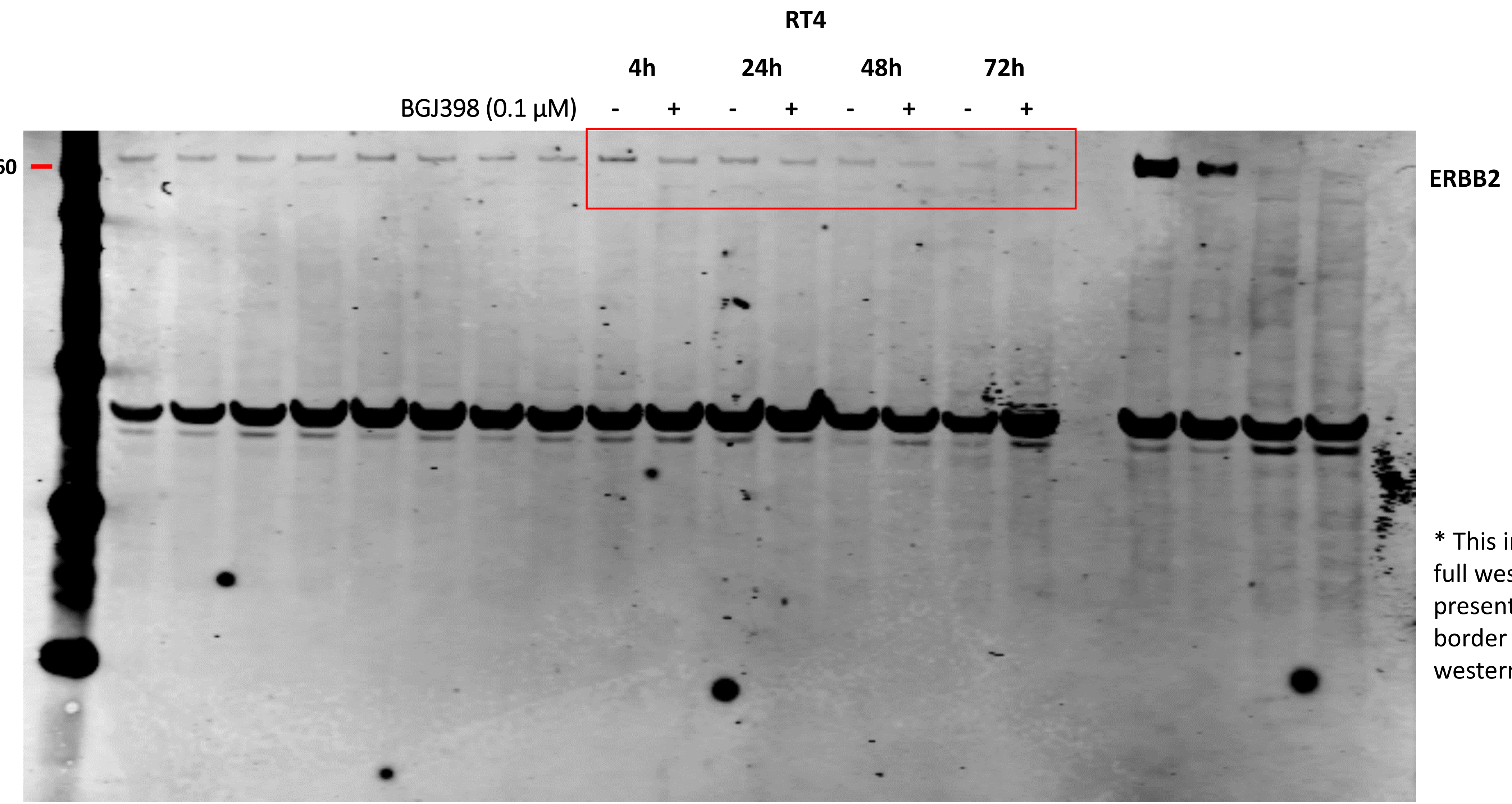

* This image shown represents the full western blot. Edges were not present due to the scanned image border being smaller than the western membrane.

Fig 4

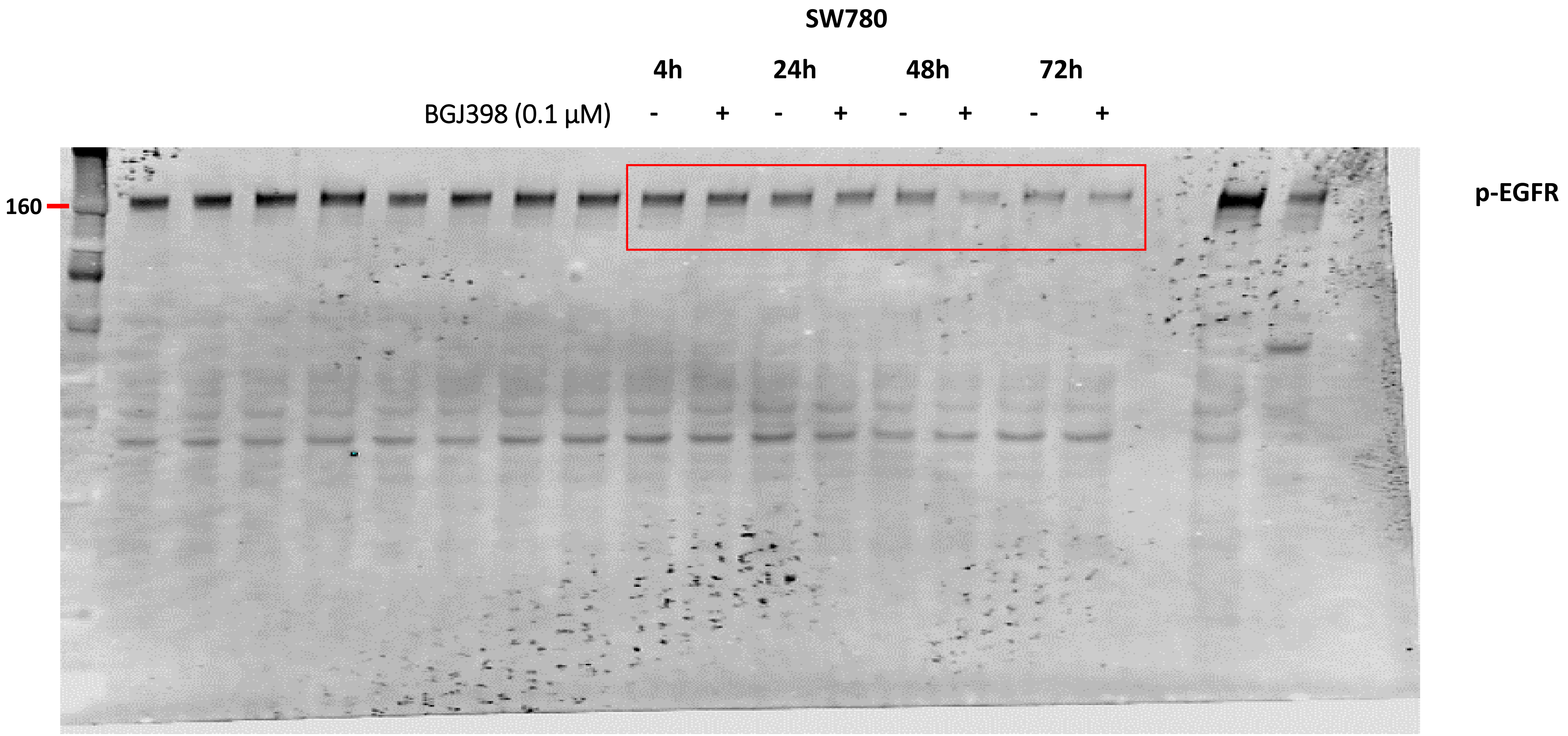

Fig 4

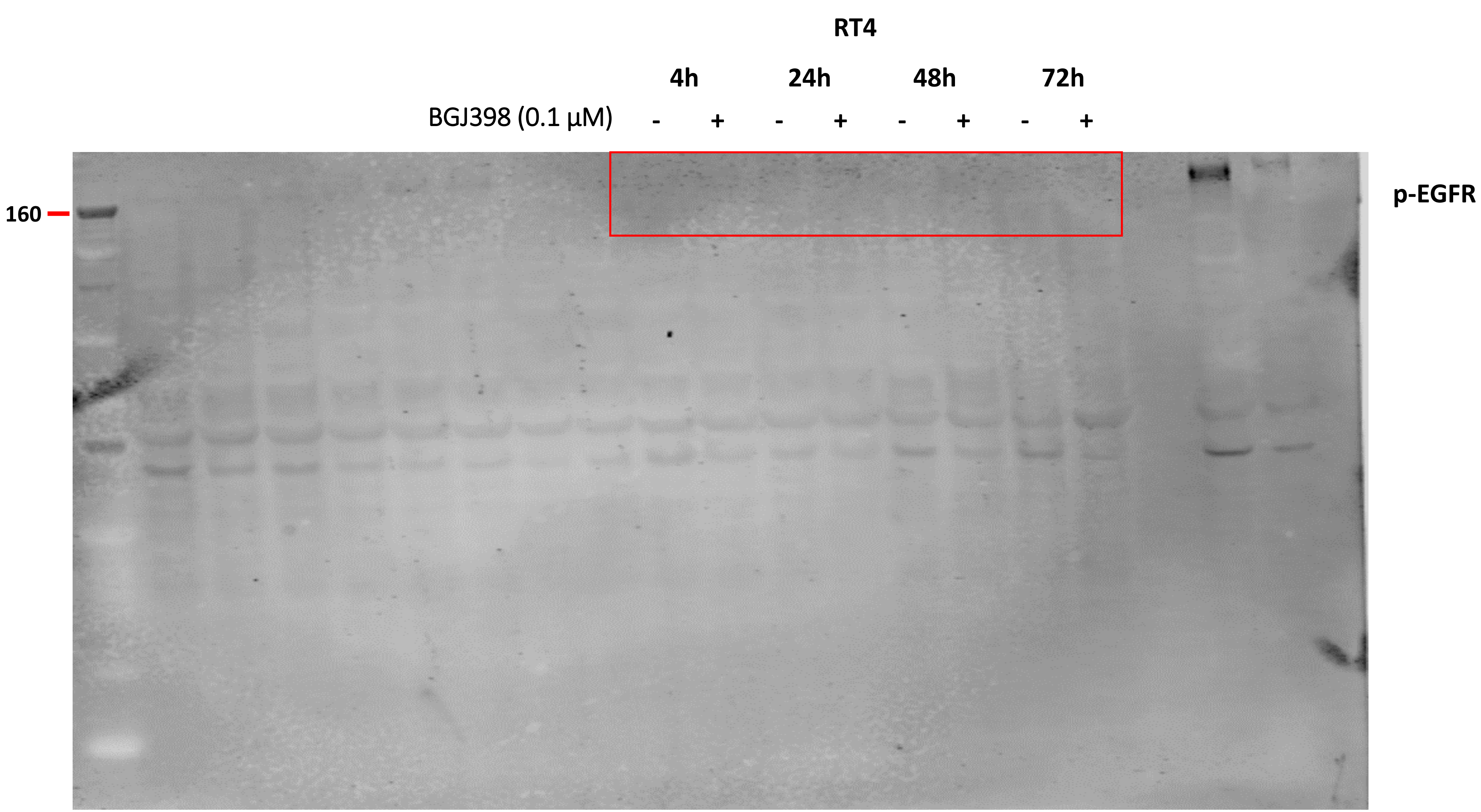

Fig 4

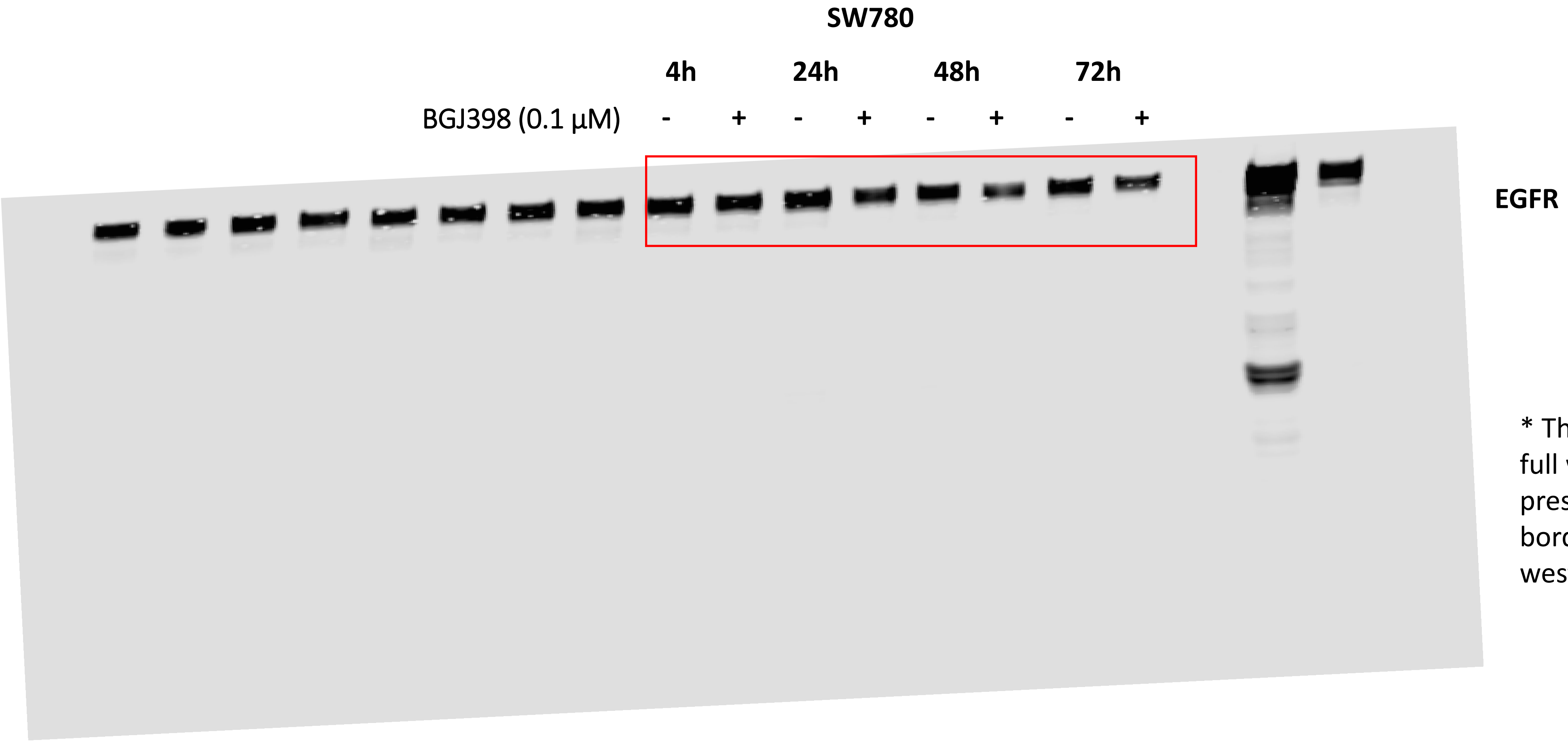

* This image shown represents the full western blot. Edges were not present due to the scanned image border being smaller than the western membrane.

Fig 4

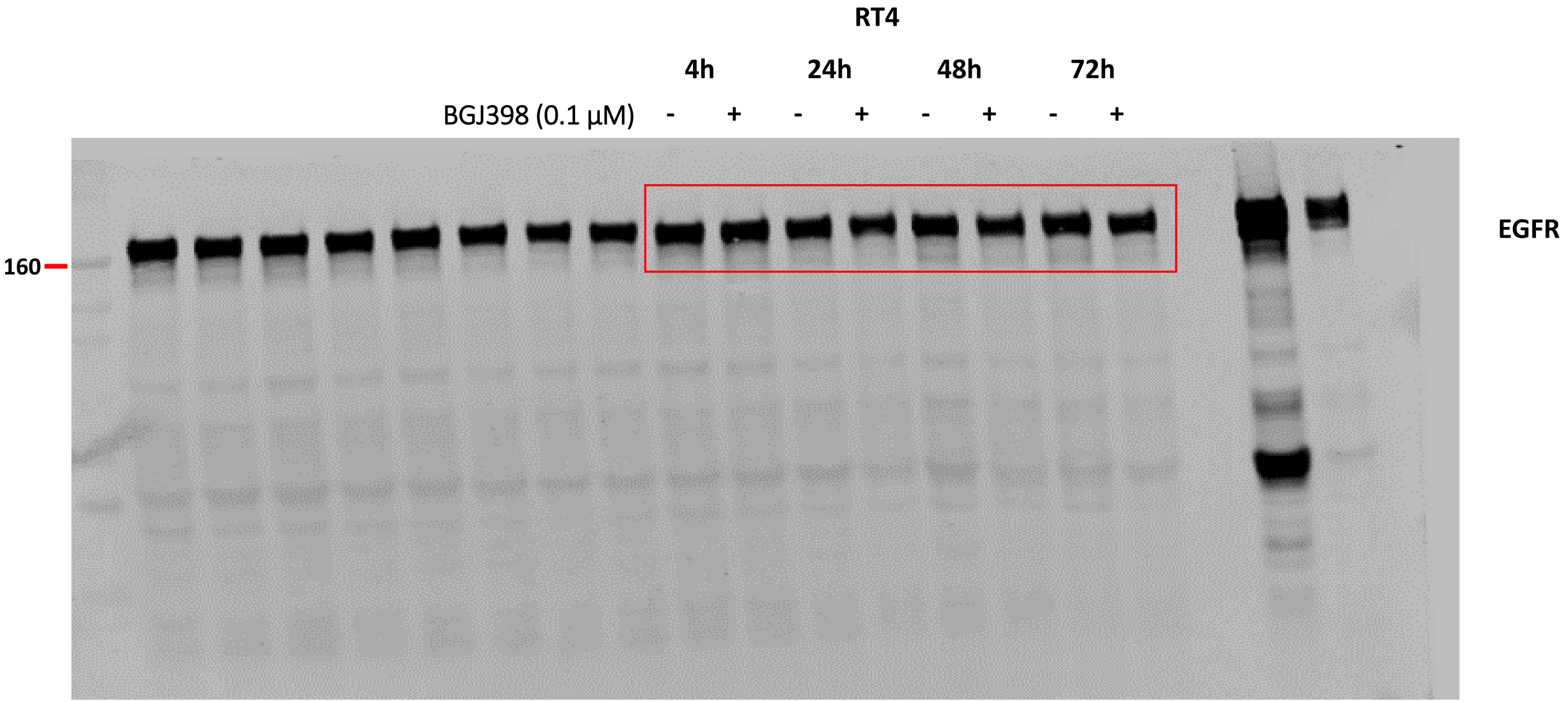

Fig 4

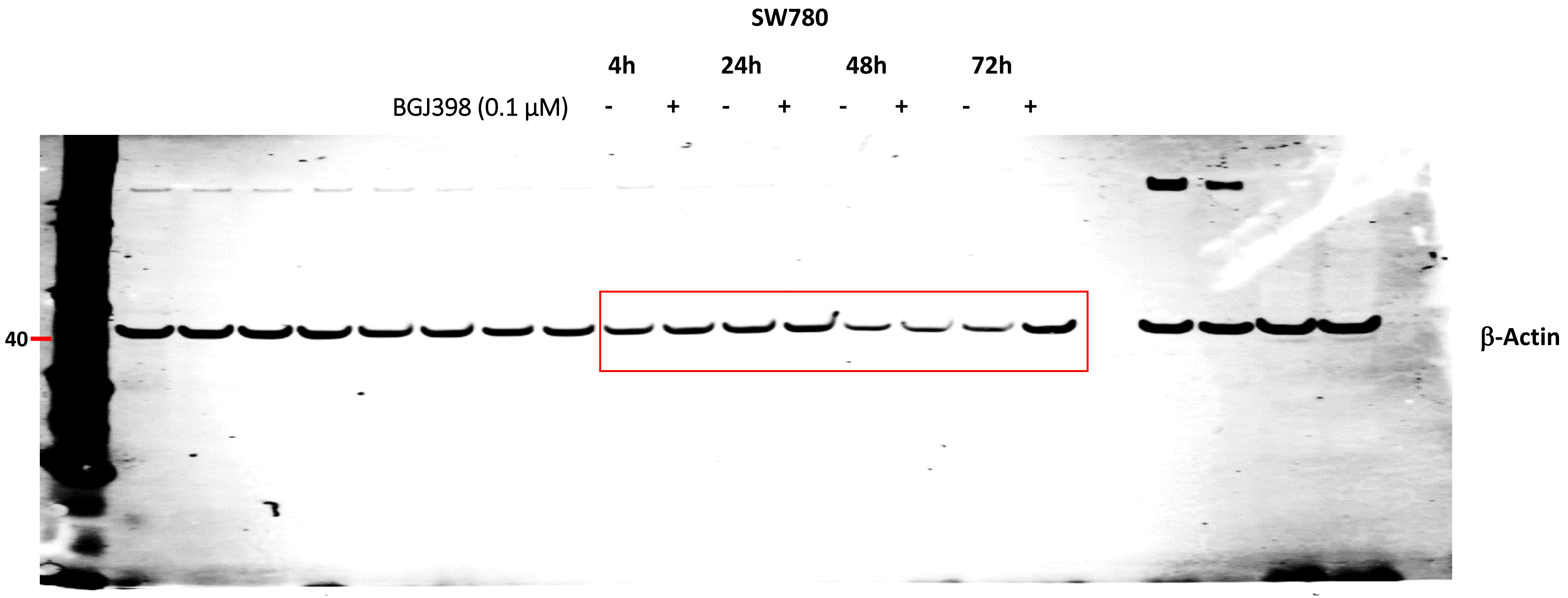

Fig 4

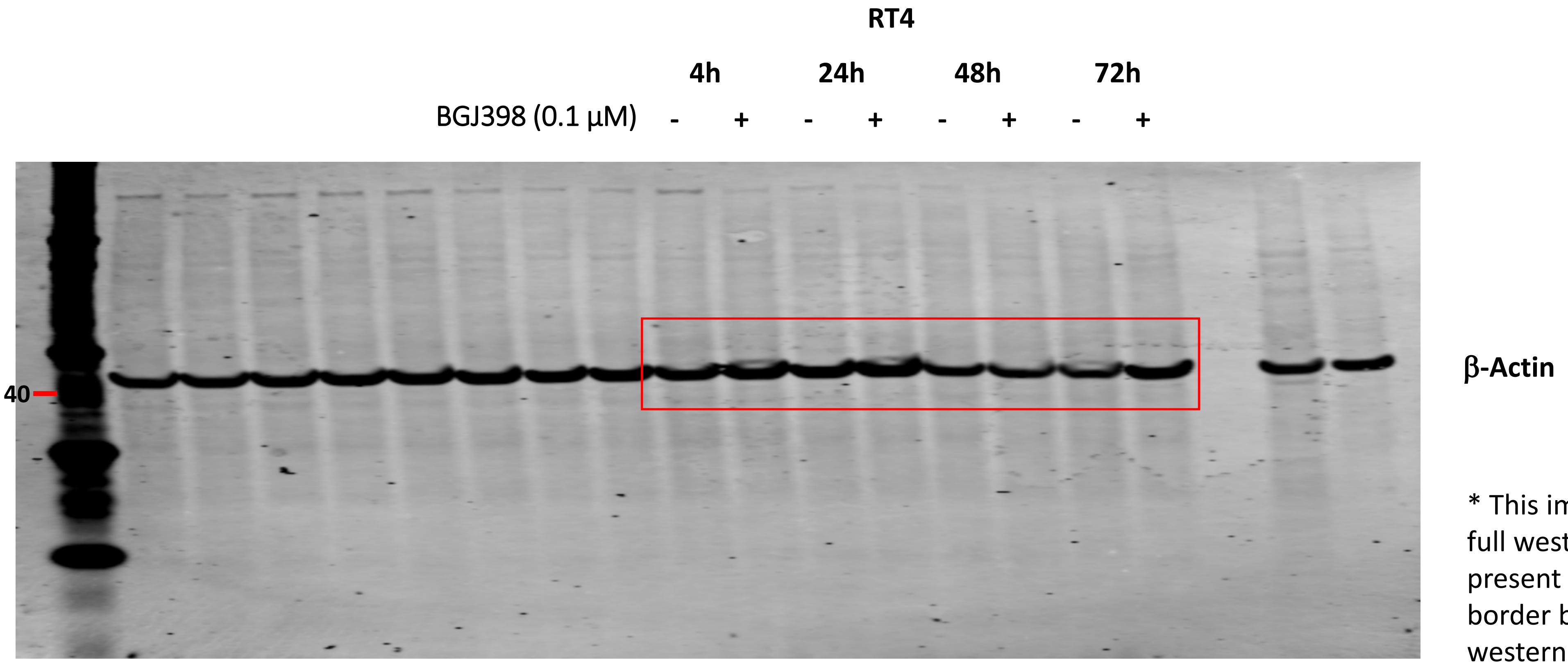

* This image shown represents the full western blot. Edges were not present due to the scanned image border being smaller than the western membrane.

Fig 5

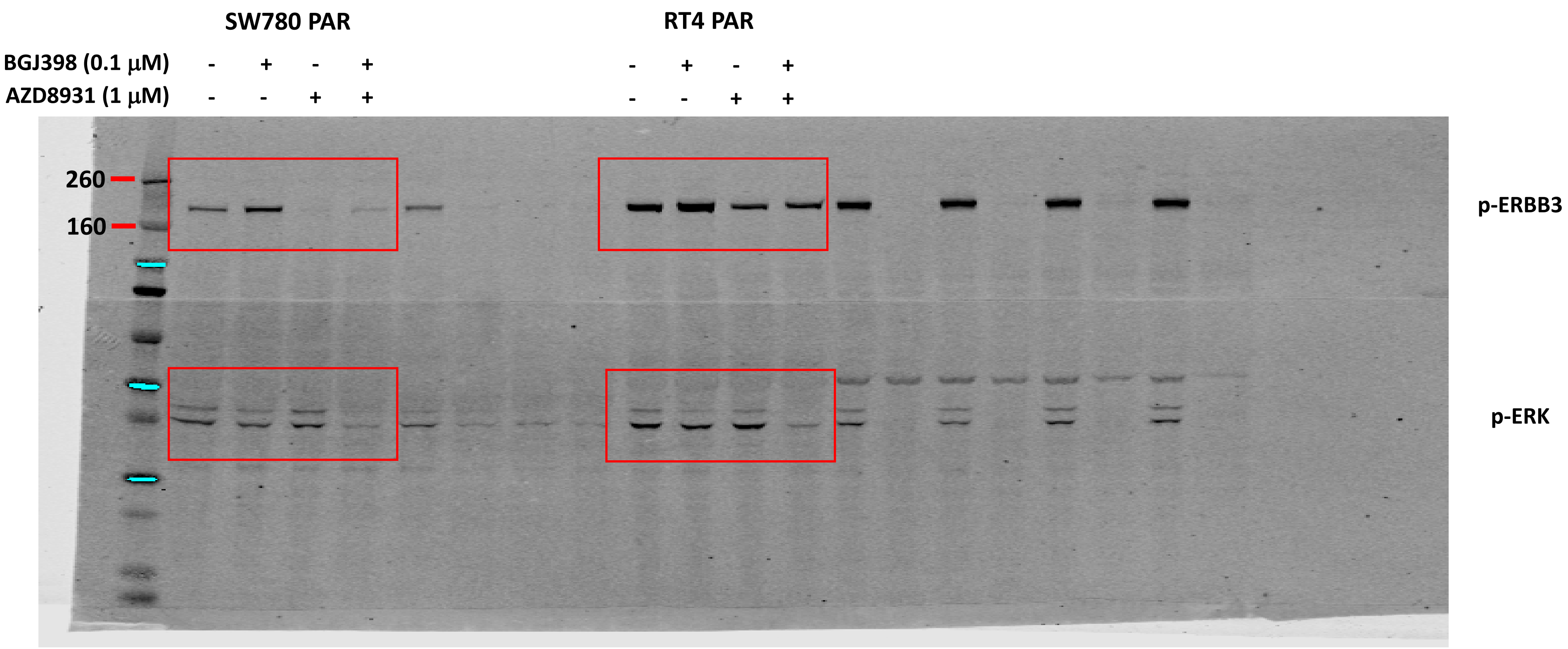
SW780 PAR
RT4 PAR
BGJ398 (0.1 μM) - + - + - + - +
AZD8931 (1 μM) - - + + - - + +
260
160
p-ERBB3
p-ERK

Fig 5

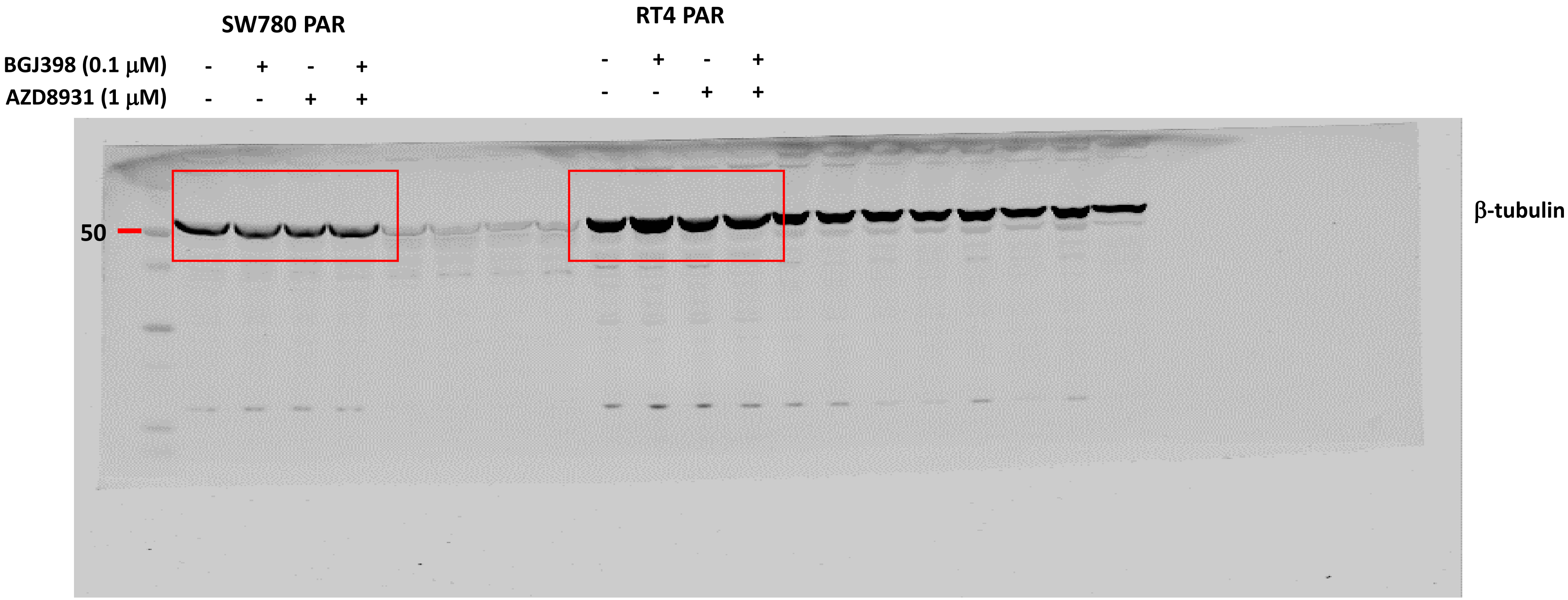

Supplement: Supplementary file 1 — Additional file 1. [file 12885_2022_9478_MOESM1_ESM.pdf]
